# Spiral-Wave Dynamics in a Mathematical Model of Human Ventricular Tissue with Myocytes and Fibroblasts

**DOI:** 10.1371/journal.pone.0072950

**Published:** 2013-09-04

**Authors:** Alok Ranjan Nayak, T. K. Shajahan, A. V. Panfilov, Rahul Pandit

**Affiliations:** 1 Centre for Condensed Matter Theory, Department of Physics, Indian Institute of Science, Bangalore, India; 2 Centre for Nonlinear Dynamics in Physiology and Medicine, McGill University, Montreal, Canada; 3 Department of Physics and Astronomy, Gent University, Gent, Belgium; 4 Jawaharlal Nehru Centre for Advanced Scientific Research, Bangalore, India; Georgia State University, United States of America

## Abstract

Cardiac fibroblasts, when coupled functionally with myocytes, can modulate the electrophysiological properties of cardiac tissue. We present systematic numerical studies of such modulation of electrophysiological properties in mathematical models for (a) single myocyte-fibroblast (MF) units and (b) two-dimensional (2D) arrays of such units; our models build on earlier ones and allow for zero-, one-, and two-sided MF couplings. Our studies of MF units elucidate the dependence of the action-potential (AP) morphology on parameters such as 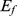, the fibroblast resting-membrane potential, the fibroblast conductance 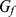, and the MF gap-junctional coupling 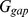. Furthermore, we find that our MF composite can show autorhythmic and oscillatory behaviors in addition to an excitable response. Our 2D studies use (a) both homogeneous and inhomogeneous distributions of fibroblasts, (b) various ranges for parameters such as 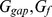, and 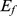, and (c) intercellular couplings that can be zero-sided, one-sided, and two-sided connections of fibroblasts with myocytes. We show, in particular, that the plane-wave conduction velocity 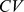 decreases as a function of 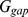, for zero-sided and one-sided couplings; however, for two-sided coupling, 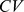 decreases initially and then increases as a function of 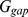, and, eventually, we observe that conduction failure occurs for low values of 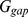. In our homogeneous studies, we find that the rotation speed and stability of a spiral wave can be controlled either by controlling 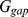 or 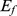. Our studies with fibroblast inhomogeneities show that a spiral wave can get anchored to a local fibroblast inhomogeneity. We also study the efficacy of a low-amplitude control scheme, which has been suggested for the control of spiral-wave turbulence in mathematical models for cardiac tissue, in our MF model both with and without heterogeneities.

## Introduction

Cardiac fibroblasts, which are connective, non-myocyte cells, play a major role in producing myocyte cells, both in the early stage of heart development and after a myocardial infarction. Experimental studies [1], [2] suggest that such fibroblasts can be coupled functionally with myocytes, under both physiological and pathophysiological conditions. Fibroblasts can, therefore, modulate the electrophysiological properties of cardiac tissue. However, it is not clear yet what range of values we should use for the gap-junctional conductance 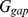 of a fibroblast-myocyte gap junction [3]–[5]; in intact tissue 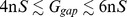 [3] and, in cell-culture preparations, 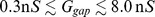 [5]. The structural organization of fibroblast cells in cardiac tissue, which consists of myocyte and non-myocyte cells (e.g., fibroblasts), is still being explored [2], [6]–[8] for different mammalian hearts. This lack of detailed structural and functional information makes it difficult to use experimental studies to uncover the precise role that fibroblasts play in the propagation of electrical impulses and spiral waves of electrical activation in cardiac tissue. Therefore, computational studies are beginning to play an important role in the investigation of the properties of mathematical models for cardiac tissue that include myocytes and fibroblasts and a coupling between them; some of these study a single, composite myocyte-fibroblast cell [9]–[12]; others have considered electrical-wave propagation in one- and two-dimensional, mathematical models for cardiac tissue, in which the fibroblasts are modelled as passive cells [11], [13], [14]. Here, we build on mathematical models that couple cardiac myocytes and fibroblasts at the single-cell level (this yields a myocyte-fibroblast MF composite) to develop a mathematical model for a two-dimensional (2D) sheet of cardiac myocytes coupled to a similar sheet of fibroblasts. Our model uses the state-of-the-art ionic model for human cardiac myocytes due to ten Tusscher, Noble, Noble, and Panfilov (TNNP) [15]; we include connections between myocytes and fibroblasts via gap junctions; and we also allow for the possibility of studying *zero-sided, one-sided*, and *two-sided* couplings (see section on “Model and Methods”).

We begin with a summary of the principal results of our extensive numerical studies, which we have designed to elucidate electrophysiological properties in mathematical models for (a) single myocyte-fibroblast (MF) units and (b) two-dimensional (2D) arrays of such units. Our studies of MF units yield the dependence of the action-potential (AP) morphology on parameters such as 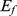, the fibroblast resting-membrane potential, the fibroblast conductance 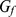, the number 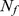 of fibroblasts coupled to a myocyte, and the MF gap-junctional coupling 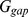; and our MF composite can show autorhythmic and oscillatory behaviors in addition to an excitable response. We also present ionic mechanisms that are responsible for the modulation of the AP as we alter 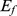 or 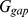. Our 2D studies use (a) both homogeneous and inhomogeneous distributions of fibroblasts, (b) various ranges for parameters such as 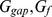, and 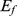, and (c) zero-, one-, and two-sided MF connections. We show that the plane-wave conduction velocity 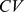 decreases as a function of 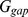, for zero- and one-sided couplings; however, for two-sided coupling, 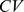 decreases initially and then increases as a function of 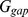, and, eventually, we observe that conduction failure occurs for low values of 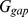. In our homogeneous studies, we find that the rotation speed and stability of a spiral wave can be controlled either by controlling 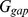 or 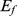. And we show that a spiral wave can get anchored to a local fibroblast inhomogeneity. We demonstrate the efficacy of a low-amplitude control scheme, which has been suggested for the control of spiral-wave turbulence in mathematical models for cardiac tissue [16]–[19], in our MF model both with and without heterogeneities. We include several animations of our simulations (Videos S1–S12) to provide a quick, pictorial overview of our results.

We now present a brief and illustrative overview of some earlier studies that have investigated the AP morphological behavior at the level of MF units to determine the effects of the extra electrical load, either because of passive or active fibroblasts, in both animal- and human-ventricular-cell models; we also discuss the propagation of electrical impulses in cardiac tissue models with fibroblasts. In Table 1 we give the ranges of parameters that have been used, in a variety of experimental and computational studies, of MF composites.

**Table 1:** Cardiac fibroblast parameter values used in various experimental and computational studies.

References	Type of studies	Type of fibroblast	Parameter ranges
Rook, *et al.* [5]	in culture	rat	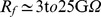
			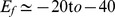 mV
			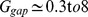 nS
Kohl, *et al.* [3]	in vitro	rat	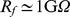
	and		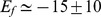 mV
	in culture		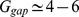 nS
Kiseleva, *et al.* [46]	in vitro	rat	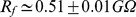 (control case)
			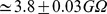 (diseased case)
			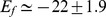 mV (control case)
			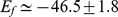 mV (diseased case)
Kamkin, *et al.* [47]	in vitro	human	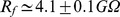
			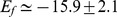 mV
Kamkin, *et al.* [48]	in vitro	rat	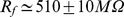 (control case)
			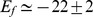 mV (control case)
			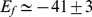 mV to 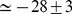 mV (diseased case)
Chilton, *et al.* [49]	in culture	rat	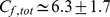 pF
			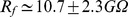
			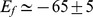 mV (for 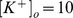 mM)
			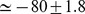 mV (for 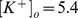 mM)
Shibukawa, *et al.* [50]	in culture	rat	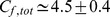 pF
			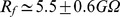
			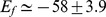 mV
Xie, *et al.* [9]	computational	passive	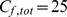 pF
			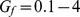 nS
			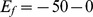 mV
			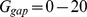 nS
			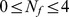
Sachse, *et al.* [10]	computational	active	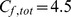 pF
			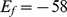 mV
			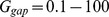 nS
			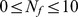
Jacquemet, *et al.* [11]	computational	active	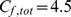 pF
			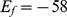 mV
			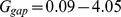 nS
			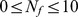
MacCannell, *et al.* [12]	computational	active	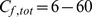 pF
			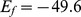 mV
			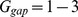 nS
			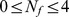

Xie, *et al.* [9] have used two different ionic models for myocytes, namely, the Luo-Rudy Phase 1 (LRI) model [20], with modified maximal conductances, and a rabbit-ventricular-cell model [21], coupled to models of passive and active fibroblasts via a gap-junctional conductance (Table 1). In their passive-fibroblast studies, for low values of 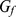, they have found that the action-potential duration (APD) is always prolonged relative to its value APD^m^ for an uncoupled myocyte; however, if 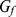 is large, then the APD is less than APD^m^, if 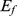 is low, but greater than APD^m^, if 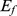 is high. They have obtained similar results in models with active fibroblasts.

Sachse, *et al.* [10] have shown, with a rat-ventricular-cell model [22], that the APD is prolonged relative to APD^m^ in an active-fibroblast model. Their study shows that the myocyte APD, measured at 90% repolarization, increases from 38.9 ms to 61.3 ms if 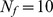 and 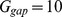 nS; however, if 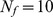 and 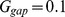 nS, this APD decreases from 39.0 ms to 37.1 ms. Their studies also show that the myocyte resting membrane potential, 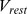, and the maximal upstroke velocity, 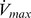, depend on 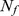 and 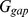.

Jacquemet, *et al.* [11] have studied a mouse-ventricular-cell model [23] coupled to a simple fibroblast model that includes a delayed activation of the membrane current. Their study has revealed that the myocyte APD is prolonged from 14.4 ms to 14.8 ms, its action potential amplitude (APA) reduced from 115.1 mV to 114.6 mV, and there is a slight elevation of the resting membrane potential 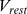 from −82.3 mV to −82.0 mV, when a single fibroblast is coupled to a myocyte with 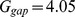 nS. They have also studied the dependence of the APA, APD, 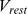, and 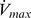 on 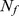 and 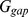 by measuring them at a site of a myocyte cell, located in the middle of a cable, which contains 50 myocyte cells covered by a layer of fibroblasts. They have found, e.g., that (a) the APA, APD, and 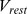 change to 93.1 mV, 19.3 ms, and −80.5 mV, respectively, from their corresponding uncoupled values 100.8 mV, 15.7 ms, and −82.3 mV, when 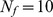 and 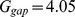 nS, and (b) 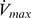 changes to −68 mV/ms from its uncoupled value −92 mV/ms, when 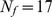 and 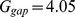 nS.

MacCannell, *et al.* [12] have considered fibroblast models, principally active but also passive, coupled to a human-ventricular-myocyte model [15]. They have presented representative results for a single MF unit for the passive case; they have found that the myocyte APD *increases* from its uncoupled value 263 ms to 273 ms or 275 ms for 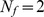 and 4, respectively. By contrast, in their active-MF model, they have found that the APD *decreases* from 263 ms to 195 ms and 155 ms, respectively, when 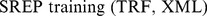 or 4 (with the above-mentioned parameter values); furthermore, 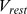 is elevated from its uncoupled value −86.1 mV to −85.8 mV, if 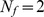, and −85.3 mV, if 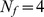; and the APD shortening can be enhanced by increasing either 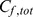 or 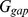; e.g., if 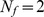 the APD decreases from 263 ms to 225 ms or 207 ms, respectively, for 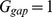 and 2 nS, with 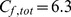 pF, 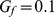 nS, and 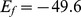 mV. This study also obtains similar results when it holds all parameters at the values given above but uses 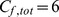 pF or 63 pF.

Both in cell culture and in intact tissue, fibroblasts can couple functionally to adjacent myocytes via a gap junction at the single-cell level by expressing either the Cx43 or the Cx45 gap-junction protein or connexin. Miragoli, *et al.* [24] have shown the expression of connexins, between fibroblasts and, at contact sites, between fibroblasts and cardiomyocytes, by studying cocultured fibroblasts coated over rat-ventricular-myocyte strands; and Gaudesius, *et al.* [25] have reported that Cx43 and Cx45 are expressed among fibroblasts and between fibroblasts and myocytes when fibroblasts are inserted in cocultures of neonatal rat-heart cells in a monolayer.

Fibroblasts can play a major role in the propagation of electrical impulses in cardiac tissue. Some *cell-culture* [13], [24]–[26] and *in-silico* [11], [13], [14] studies have reported the suppression of impulse propagation in cardiac tissue because of fibroblasts. For example, Miragoli, *et al.* [24] have studied electrical-impulse propagation in cultured strands of myocytes coated by fibroblasts and shown that the conduction velocity 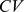 decreases by an amount that depends on the density of fibroblasts. The work of Gaudesius, *et al.* [25] has demonstrated that conduction delay occurs because of the insertion of fibroblasts between myocytes in cultured myocyte strands; the delay depends on the number of inserted fibroblasts; and finally conduction block occurs when the length of the inserted fibroblasts exceeds 300 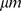. Zlochiver, *et al.* [13] have studied the propagation of electrical impulses in a monolayer of myocytes and fibroblasts of neonatal rats; in one set of experiments they have either increased or decreased the gap-junction coupling by overexpressing Cx43 or by using silencing RNAi; in another set of experiments they have varied the ratio of fibroblasts to myocytes. In the former case, they have observed that an increase in the gap-junctional conductance first leads to a decrease in 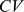 and then an increase; in the second set of experiments they have found that 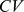 decreases as the fibroblast density increases. McSpadden, *et al.* [26] have studied electrical-wave propagation in a monolayer of neonatal rat cardiac myocytes electrotonically loaded with a layer of cardiac fibroblasts; they have used an optical-mapping technique to find the dependence of such impulse propagation on the gap-junctional conductance 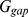; and they have found that impulse propagation, in both the transverse and longitudinal directions, changes significantly when fibroblasts are loaded on the myocyte monolayers; e.g., as the fibroblast coverage area increases from the 0–15% coverage range to the 75–100% coverage range, the conduction velocity 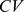, in loaded monolayers, decreases from 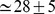 cm/s to 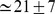 cm/s, in the longitudinal direction, and from 13±3 cm/s to 9±3 cm/s, in the transverse direction.

Xie, *et al.* [14] have followed Ref. [27] to model MF tissue in three different ways, namely, with (a) zero-sided, (b) single-sided, and (c) double-sided connections, by using the LRI [20] ventricular-cell model for myocytes with slight modifications of the original parameters. In their zero-sided connection model, passive fibroblasts are inserted in a 2D layer of myocytes; but they are functionally uncoupled with myocytes at their contact sites, so the fibroblasts are equivalent to conduction inhomogeneities [16], [17], [28]–[30]. In the single-sided connection model, connected fibroblasts are loaded on the top of a 2D layer of myocytes; therefore, they are equivalent to an extra, local electrical load. In the double-sided-connection model, connected fibroblasts are inserted in a 2D myocardial layer, with myocytes and fibroblasts connected at contact sites; this provides an additional conduction pathway for electrical signals, so the fibroblasts are qualitatively similar to ionic inhomogeneities [16], [17], [31]. Their studies of fibroblasts randomly attached on the top of a 2D myocyte sheet (i.e., single-sided connections), show that, for low fibroblast membrane conductances 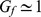 and with the fibroblast resting-membrane potential 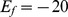 mV, 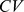 initially remains almost unchanged as the fibroblast-myocyte (FM) ratio increases; but then it decreases quickly as the FM ratio approaches 3. If, however, 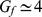, 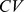 increases initially and then decreases rapidly as the FM ratio approaches 1. However, in both cases, with low and high values of 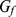, conduction failure occurs when 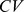 decreases to 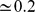 m/s from its uncoupled value 0.56 m/s. Furthermore, when 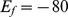 mV (i.e., close to the myocyte resting-membrane potential), 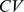 decreases linearly from 0.56 m/s to 0.49 m/s as the FM ratio increases from 0 to 3; this trend is almost independent of the value of 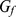. They have also studied the effects of the random insertion of fibroblasts in a 2D sheet of myocytes sheet; the resulting myocyte-fibroblast pairs can have zero-sided or double-sided connections. When fibroblasts are inserted in series, 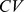 decreases almost linearly as the FM ratio increases, for zero-sided connections, and conduction failure occurs if the FM ratio is above 3. Similar results are observed with double-sided connection when fibroblasts, with 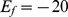 mV and a low value of 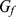 (1 nS) are coupled with myocytes. However, for larger values of 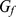 (4 nS), 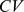 decreases much faster as the FM ratio increases, and conduction failure occurs if the FM ratio is below 1. Furthermore, if 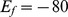 mV, 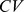 is only slightly different from that with uncoupled fibroblasts and almost independent of 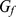. If the fibroblasts lie parallel to myocytes in a 2D sheet, they have found that, with random laterally inserted fibroblasts coupled to all neighboring cells (double-sided connection), 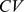 changes in both longitudinal and transverse directions, but to a different extents. In the longitudinal direction, 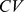 is similar to that in models with random fibroblast attachment; however, in the transverse direction, 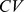 decreases much more rapidly, as in to models with random fibroblast insertions. These authors have also studied the effects of 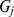 on 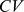 for randomly inserted fibroblasts (double-sided connection) in a 2D sheet of myocytes. For low 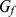 (0.1 nS), with an FM ratio of 1, they have found that 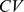 decreases first and then increases as 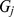 increases (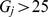 nS). However, for high 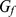 (2 nS), 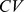 increases to a maximum (at 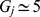 nS), then decreases to minimum (at 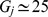 nS), and eventually increases linearly as 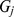 increases.

Zlochiver, *et al.* [13] have studied impulse propagation, by inserting fibroblasts, in a 2D sheet of myocyte tissue in the dynamic Luo-Rudy (LRd) [32], [33] model of a mammalian ventricular cell [34]. Their studies show that 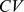 first increases and then decreases as 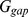 increases and then decreases as a function of fibroblast-myocyte area ratio, in agreement with their experimental observations.

The remaining part of this paper is organized as follows. In the Section on “Model and Methods” we describe the formulation of our myocyte-fibroblast model, for a single cell and for 2D tissue; we also describe the numerical schemes that we use to solve the model equations. In the Section on “Results” we present the results of our numerical calculations. In the Section on “Discussion and Conclusion” we discuss the significance of our results and compare them with results from other experimental and computational studies.

## Model and Methods

In this Section, we build on earlier mathematical models for (a) cardiac tissue [15] and (b) the coupling, at the level of single cells, of cardiac myocytes and cardiac fibroblasts [9]–[12] to develop a mathematical model for a 2D sheet of cardiac myocytes coupled to a similar sheet of fibroblasts. We use the ionic model for human cardiac myocytes [15] due to ten Tusscher, Noble, Noble, and Panfilov (TNNP); we include connections between myocytes and fibroblasts via gap junctions; and we also allow for the possibility of studying *zero-sided, one-sided*, and *two-sided* couplings as illustrated in the schematic diagram of Fig. S1 in Material S1.

The cell membrane of a cardiac myocyte is modelled by the following ordinary differential equation (ODE) [35], [36]

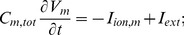
(1)here 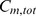 is the total cellular capacitance, 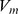 is the transmembrane potential, i.e., the voltage difference between intra- and extra-cellular spaces, 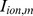 is the sum of all ionic currents that cross the cell membrane, and 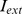 is the externally applied current. Similarly, the membrane potential of a passive fibroblast is given by the ODE
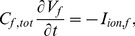
(2)where 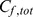, 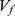, and 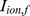 are, respectively, the total cellular capacitance, the transmembrane potential, and the sum of all ionic currents for the fibroblast. The passive nature of the fibroblasts allows us to write
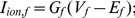
(3)here 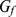 and 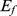 are, respectively, the conductance and the resting membrane potential for the fibroblast. If a single myocyte cell is coupled with 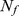 fibroblasts via the gap junctional conductance 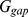, its transmembrane potential can be modelled by the following set of equations:



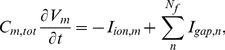
(4)

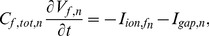
(5)where
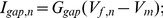
(6)here 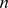 labels the fibroblasts that are connected to the myocyte via 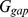; note that 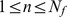 and, for the identical fibroblasts we consider here, 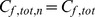, 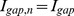, and 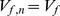, for all 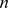. The physical units that we use for our model are as follows: time 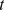 is in ms, the transmembrane potentials 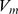 and 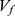 are in mV, the transmembrane currents 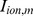 and 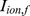 are in pA, therefore, current densities for the myocyte are in pA/pF, the total cellular capacitances 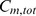 and 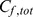 are in pF, and the fibroblast conductance 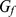 and the gap-junctional conductance 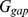 are in nS.

As suggested in Ref. [37], the dynamics of 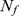 identical fibroblasts coupled to a myocyte is equivalent to the dynamics of a single fibroblast coupled to a myocyte with coupling strength, 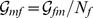, where 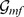 is the coupling strength of a myocyte to 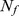 fibroblasts and 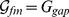 is the coupling strength of a fibroblast to a myocyte. Therefore, we have performed simulations by using only one fibroblast per myocyte in our 2D simulation domain. This is equivalent to a myocyte being coupled with 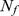 fibroblasts with coupling strength 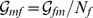. Furthermore, in our 2D model, the maximum number of fibroblasrs 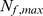 allowed per site is roughly related with the ratio of 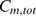 and 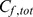 because they are related to the surface area of the cell; in experiments, 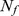 depends on the ratio of these surface areas and the volume fractions of myocytes and fibroblasts. These considerations are important because fibroblasts are considerably smaller than myocytes as we discuss in greater detail in the section on “Discussion and Conclusion”.

In our 2D computational studies, we use a simulation domain in which we have one layer of fibroblasts on top of a myocyte layer as illustrated in Fig. S1 in Material S1. Such a simulation domain is motivated by the experiments of Refs. [6]–[8], [38]. We model this myocyte-fibroblast bilayer by using the following discrete equations [39], [40]:
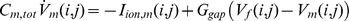


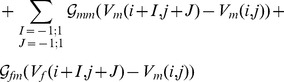
(7)

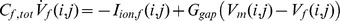


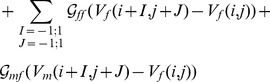
(8)here the dots above 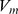 and 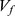 denote time derivatives, 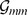 and 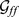 represent, respectively, intercellular couplings in the myocyte and fibroblast layers; and 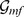 and 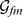 account for cross couplings between myocyte and fibroblast layers; if one of the cross-coupling coefficients, say 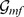, is nonzero, then the other, 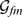, must also be nonzero to ensure current conservation; myocyte and fibroblast composites are coupled at a given site by 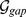; in addition, we allow for intercellular couplings (see Fig. S1 in Material S1) that can be categorized naturally as follows: (A) zero-sided: 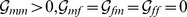; (B) one-sided: 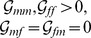; and (C) two-sided: 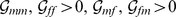; the index (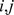) refers to the cell associated with the node under consideration; the conductances 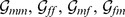, and 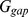 are in nS. Given that fibroblasts are much smaller than myocytes, the terms 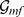, 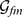, and 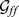 are negligible relative to 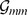 unless a cluster of 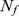 fibroblasts is formed, with 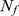 large enough for the size of the cluster to be comparable to that of a myocyte; such clusters can couple to myocytes and to each other and lead to a realization of the one- and two-sided models mentioned above (see Fig. S1 in Material S1; and for a schematic diagram of connections between a myocyte and a clusters of 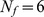 fibroblasts see Fig. 7 in Ref. [37]); in such an effective model, we assume, at the level of the simplest approximation, that the fibroblasts in a cluster interact only to the extent that they form the cluster.

We use a 2D square domain consisting of 600×600 grid points and lattice spacing 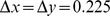 mm, so the side of each square domain is 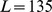 mm; one of these layers contains myocytes and the other fibroblasts as shown in Fig. S1 in Material S1. These two layers are separated by a distance 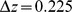 mm. We use a forward-Euler method for the time evolution of the transmembrane potentials with a time step 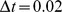 ms. We use *no-flux* (Neumann) boundary conditions on the edges of the simulation domain. The initial condition we use is related to the one given in Ref. [17]; we describe it in detail in subsection 2 in the section on “Results”.

It is often useful to track the trajectory of the tip of a spiral wave to investigate the stability of a spiral, its transitions, and its rate of drifting in a 2D simulation domain. The tip of such a spiral wave is normally defined as the point where the excitation wave front and repolarization wave back meet; this point can be found as the point of intersection of an isopotential line, of constant membrane potential, 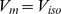 (in general 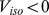 mV), and the line 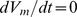 [15], [41], [42]. Another classical technique, which tracks the spiral-wave tip in a two-variable model, obtains this tip by finding the point where the isocontours of the two state variables intersect [42]–[45]; this technique can also be used in a complex mathematical model for cardiac tissue provided the model has at least one slow and one fast variable. We have developed a tip-tracking algorithm that locates the tip position, i.e., the point at which the wave-front and wave-back meet each other, by monitoring 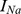, the sodium current. This is the predominant current in the depolarization phase of the AP and is, therefore, responsible for depolarizing the cells that lie ahead of the wave front, in the 2D simulation domain; thus, it plays an important role in the spatiotemporal evolution of this wave front. Hence, we find the minimum strength of 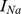 that can yield an AP; and we use this as a reference value to track the tip position. Given the sharpness of the depolarization, pseudocolor plots of 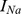 show a fine line along a spiral-wave arm (see, e.g., Fig. 2A in Ref. [16]); this fine line stops at the spiral tip and provides, therefore, an accurate way of tracking the spatio-temporal evolution of this tip.

## Results

In our previous studies [16], [17], we have investigated the interaction of a spiral-wave with conduction and ionic inhomogeneities in the TNNP model for cardiac tissue. Here we elucidate spiral-wave dynamics in the presence of fibroblasts by using the mathematical model we have developed in the section on “Model and Methods”. In subsection 1.1 we present results for the morphology of the action potential (AP) in a myocyte-fibroblast (MF) composite; in particular, we examine the dependence of the AP on 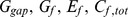, and 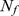. Subsection 2 contains our results for spiral-wave dynamics in a homogeneous MF bilayer, in which MF composites are coupled; we consider zero-, one-, and two-sided couplings. In subsection 2 we explore the dynamics of spiral waves in a sheet of myocytes with an inhomogeneity that is an MF bilayer. The last subsection 3 examines the efficacy of the low-amplitude, mesh-based control scheme of Refs. [16], [18], [19] in the elimination of spiral waves in the homogeneous MF bilayer and the sheet of myocytes with an MF-bilayer inhomogeneity.

### 1 A Myocyte-Fibroblast (MF) Composite

Fibroblast cells act like an electrical load on myocytes. This load, which depends, principally, on the parameters 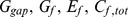, and 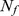, and alters the electro-physiological properties of a myocyte that is coupled to a fibroblast. In particular, it modifies the morphology of the action potential (AP). Earlier computational studies [9]–[12], which we have summarized in the “Introduction” section, have investigated mathematical models for a single unit of a myocyte and fibroblasts, for both animal and human ventricular cells and with passive or active fibroblasts. Most of these computational studies focus on the modification of the AP by (a) the number 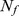 of fibroblasts per myocyte and (b) the gap-junctional conductance 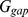. In the numerical studies that we present here we use a composite myocyte-fibroblast (MF) system with 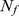 passive fibroblasts per myocyte. We examine in detail the dependence of the AP of this composite on the parameters of the model, namely, the membrane capacitance 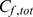, the membrane conductance 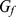, the resting membrane potential 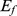, and the coupling strength 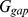; the trends we uncover are in qualitative agreement with various experiments [3], [5].

The ranges of parameters, which we use for our composite MF system, are consistent with those found in experimental studies [3], [5], [46]–[50] and those used in earlier computational studies [9]–[12] as we summarize in Table 1 and discuss in detail in Material S1. To investigate in detail the effect of fibroblasts on a myocyte, we use the following wide ranges of parameters (these encompass the ranges used in the experimental and computational studies mentioned in Table 1): 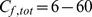 pF, 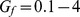 nS, 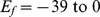 mV, and 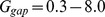 nS for our MF composites. However, to observe some special properties, such as autorhythmicity of MF composites, we vary the fibroblast parameters and gap-junctional conductances.

**Figure 1:**
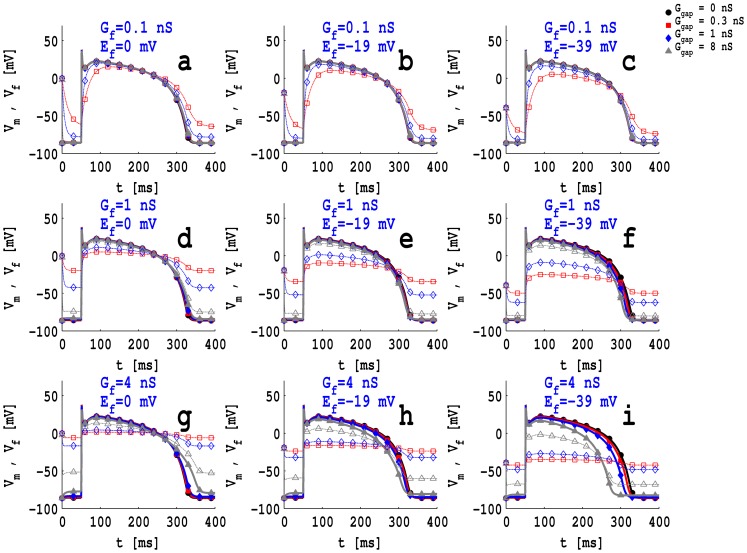
Plots of action potentials: The myocyte action potential 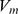 (full symbols and lines) and the fibroblast action potential 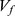 (unshaded symbols and dashed lines), with a passive fibroblast of capacitance 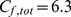 pF coupled with a myocyte for (a) 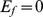 mV and 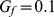 nS, (b) 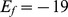 mV and 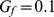 nS, (c) 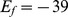 mV and 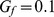 nS, (d) 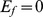 mV and 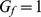 nS, (e) 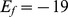 mV, and 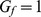 nS, (f) 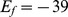 mV and 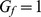 nS, (g) 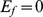 mV and 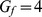 nS, (h) 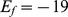 mV and 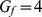 nS, and (i) 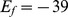 mV and 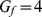 nS; red squares (full or unshaded) indicate 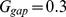 nS; blue diamonds (full or unshaded) indicate 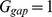 nS; gray triangles (full or unshaded) indicate 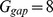 nS; black squares (full or unshaded) indicate an uncoupled myocyte.

Figures 1 (a)-(i) show plots of the myocyte transmembrane potential 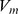 (filled symbols with solid lines) and the fibroblast transmembrane potential 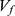 (unshaded symbols with dashed lines) versus time 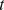, when we consider an MF composite in which a myocyte is coupled to a passive fibroblast, with 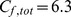 pF. In Figs. 1 (a)-(i) we use squares (▪ or 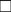) for low coupling (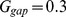 nS), diamonds (⧫ or ◊ ) for intermediate coupling (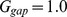 nS), triangles (▴ or ▵) for high coupling (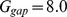 nS), and filled circles (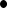) for a myocyte that is not coupled to a fibroblast. Figures 1 (a), (d), (g), which are in the first column, depict 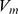 and 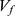 for low (0.1 nS), intermediate (1.0 nS), and high (4.0 nS) values of 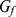 when 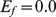 mV; their analogs for 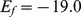 mV and −39.0 mV are given, respectively, in Figs. 1 (b), (e), (h) (second column) and Figs. 1 (c), (f), (i) (third column). These figures show the following: (i) the fibroblast action potential (fAP) is similar to the myocyte action potential (mAP) when the gap-junctional conductance 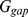 is high and the fibroblast conductance 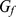 is low (Figs. 1 (a), (b), (c) in the first row); (ii) for low and intermediate values of 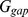 and with 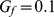 nS, the fAP plateau decreases but the APD is prolonged with respect to that of the corresponding mAP; (iii) the fAP loses its spike and notch and has a lower plateau and prolonged APD compared to the mAP when 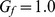 nS or 4.0 nS. Figures similar to Figs. 1 (a)-(i), but with 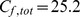 pF and 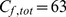, are given, respectively, in Figs. S2 (a)-(i) and Figs. S3 (a)-(i) in Material S1; these show that 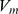 does not depend very significantly on 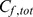 but 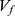 does.

**Figure 2:**
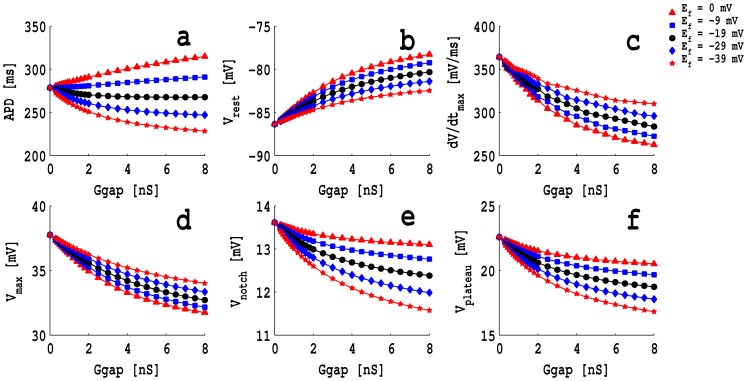
Plots of various morphological features of the myocyte action potential 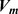 versus the gap-junctional conductance 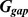; here, the myocyte is coupled with a passive fibroblast with capacitance 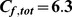 pF and conductance 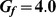 nS. (a) The action-potential duration APD versus 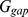; (b) the resting-membrane potential 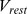 versus 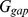; (c) the maximum upstroke velocity 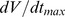 versus 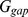; (d) the maximum value of 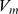, during the action potential, 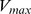 versus 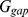; (e) the value of 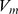 at the position of the notch, i.e., 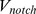 versus 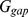; (f) the maximum value of 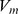, in the plateau region of the action potential, i.e., 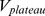 versus 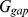; these figures show plots for the fibroblast resting membrane potential 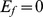 mV (full red triangles), 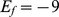 mV (full blue squares), 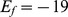 mV (full black circles), 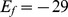 mV (full blue diamonds), and 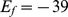 mV (full red stars).

In Figs. 2 (a), (b), (c), (d), (e), and (f) we show, respectively, plots of the action-potential duration APD, the resting-membrane potential 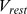, the maximum upstroke velocity 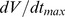, the maximum value of 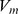, during the action potential, namely, 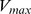, the value of 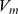 at the position of the notch, i.e., 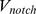, and the maximum value of 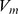, in the plateau region of the action potential, i.e., 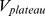 versus versus the gap-junctional conductance 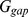; here the myocyte is coupled with a passive fibroblast with capacitance 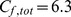 pF and conductance 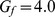 nS. These figures show plots for the fibroblast resting membrane potential 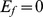 mV (full red triangles), 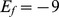 mV (full blue squares), 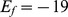 mV (full black circles), 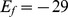 mV (full blue diamonds), and 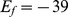 mV (full red stars). For −39mV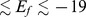 mV, the APD decreases monotonically as 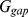 increases, but for higher values of 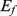, namely, −9 mV and 0 mV their is a monotonic increase of the APD with 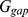. Both 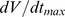 and 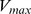 decrease monotonically as 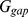 increases; the lower the value of 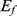, the slower is this decrease. Similarly, 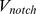 and 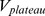 decrease monotonically as 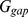 increases; but the higher the value of 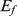, the slower is this decrease.

**Figure 3:**
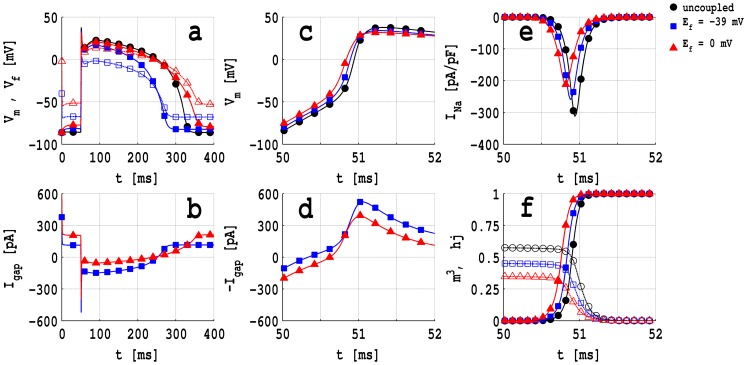
Plots of transmembrane potentials and currents for an MF composite: (a) Plots versus time 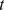 of the transmembrane potentials 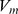 (full curves with filled symbols), for a myocyte, and 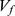 (dashed curves with open symbols), for a passive fibroblast coupled with a myocyte cell; here 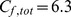 pF, 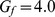 nS, 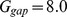 nS and 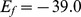 mV (blue squares) and 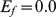 mV (red triangles); the full black curve with circles shows 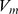 for an uncoupled myocyte. (b) Plots versus 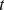 of the gap-junctional current 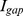 with parameters and symbols as in (b). Plots versus 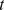, for the first 2 ms after the application of a stimulus of −52 pA/pF, of (c) 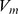, (d) 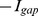, (e) the myocyte sodium current 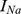, and (f) the total activation 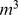 (full lines with filled symbols) and total inactivation 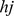 (dashed lines with open symbols) gates; the parameters and symbols here are as in (a).

In Fig. 3 (a) we present plots versus time 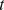 of the transmembrane potentials 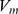 (full curves with filled symbols), for a myocyte, and 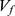 (dashed curves with open symbols), for a passive fibroblast coupled with a myocyte cell; here 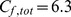 pF, 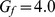 nS, 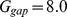 nS and 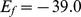 mV (blue squares) and 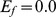 mV (red triangles); the full black curve with circles show, for comparison, a plot of 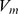 for an uncoupled myocyte. Figure 3 (b) contains plots versus 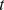 of the gap-junctional current 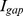 with parameters and symbols as in (b); and plots versus 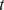 of 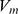, 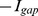, the myocyte sodium current 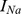, and the total activation 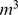 (full lines with filled symbols) and total inactivation 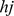 (dashed lines with open symbols) gates, for the first 2 ms after the application of a stimulus current 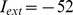 pA/pF at 50 ms for 3 ms, are depicted in Figs. 3 (c), (d), (e), and (f), respectively, for the parameters and symbols used in Fig. 3 (a). These plots show that the myocyte membrane potential 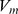 is reduced, i.e., the cell is depolarized, when a passive fibroblast is coupled with it; the larger the value of 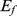, the more is the reduction in 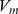. However, 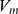 is elevated, compared to its value in the uncoupled-myocyte case, for both the values of 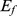 we study, because, when 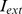 from 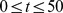 ms, the gap-junctional current 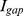 flows from the fibroblast to the myocyte as shown in Fig. 3 (b). The greater the elevation of 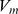 the earlier is the activation of the 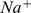 fast-activation gate 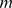, in the presence of an external applied stimulus, as can be seen by comparing Figs. 3 (c) and (f); this early activation shifts the minimum in 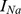 towards the left as depicted in Fig. 3 (e). Note that the product 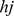, the total inactivation gating variables, decreases as 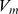 increases (dashed lines in Fig. 3 (f)) in the range 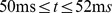. Therefore, the amplitude of 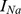 decreases with increasing 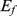, as shown in Fig. 3 (e), and leads to a reduction in the maximum rate of AP depolarization (see the plots of 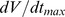 in Fig. 2 (c)); this shift in the minimum of 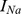 is also associated with the leftward shift of 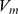 in Fig. 3 (c). A comparison of Figs. 3 (e) and (f) shows, furthermore, that, for a given value of 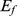, the minimum of 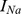 occurs at the value of 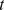 where the plots of 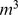 and 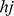 cross.

The current 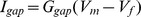 flows from the myocyte to the fibroblast or vice versa as shown in Figs. 3 (b) and (d). Before the application of a stimulus current (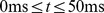 in Fig. 3 (b)), 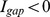, i.e., it flows from the fibroblast to the myocyte; and the current-sink capability of the fibroblast increases with 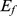, because (see Fig. 3 (a)) 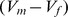 increases with 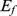. However, when we have a current stimulus 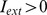, i.e., in the time interval 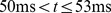, the trend noted above is reversed: the lower the value of 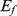 the higher is the ability of the myocyte to act as a current sink as shown in Fig. 3 (d).

Several studies [51]–[54] have shown that the contribution of individual ionic currents to the AP morphology can be examined by a partial or complete blocking of the corresponding ion channel. Therefore, we examine, for an isolated myocyte, how the AP morphology changes as we modify the major ionic currents. As in Refs. [51]–[54], we find that (a) 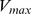 and 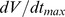 depend principally on 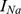, (b) 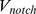 depends mainly on 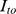, (c) the maximum of the plateau region 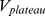 is maintained by a balance between 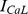 and 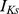, (d) the final phase of repolarization, which determines the APD, depends primarily on 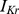 and 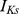, (e) the diastolic or resting phase, which decides the value of 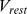, is maintained predominantly by 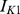, and (f) all gating variables, which determine the opening and closing of ion channels, depend on 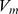, therefore, the contribution of the ionic currents to the morphology of the AP is modified as 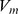 changes.

**Figure 4:**
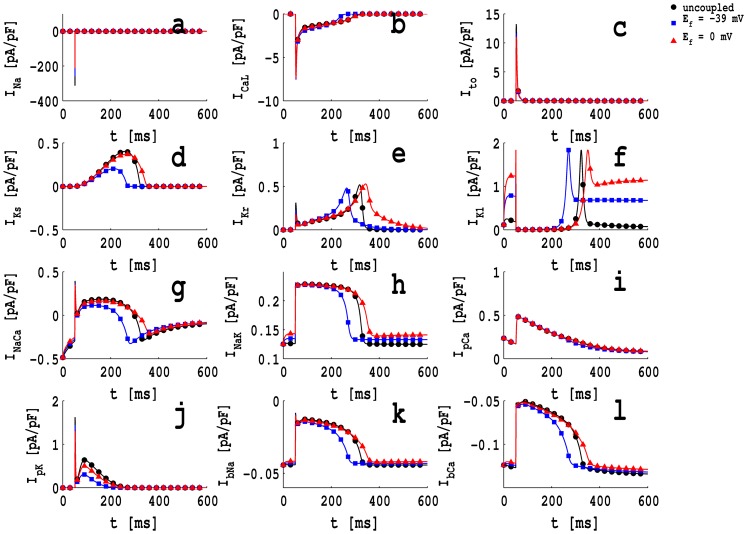
Plots of ionic current, 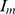, of the myocyte versus time 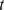 of an MF composite with 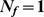; the fibroblast parameters are 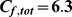 pF and 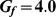 nS, and it coupled with a myocyte with 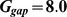 nS; the full black curve with circles shows 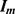 for an uncoupled myocyte; the blue filled squares and the red triangles are, respectively, for 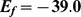 mV and 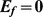
mV. (a) the fast inward 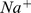 current, 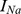; (b) the L-type slow inward 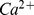 current, 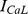; (c) the transient outward current, 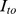; (d) the slow delayed rectifier current, 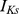; (e) the rapid delayed rectifier current, 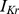; (f) the inward rectifier 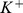 current, 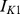; (g) the 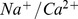 exchanger current, 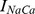; (h) the 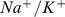 pump current, 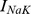; (i) the plateau 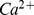 current, 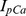; (j) the plateau 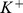 current, 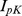; (k) the background 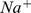 current, 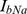; (l) the background 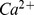 current, 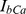.

Given these results for an isolated myocyte, we can understand qualitatively the effects on the AP morphology of a myocyte when it is coupled with a fibroblast. The coupling of a fibroblast to a myocyte modifies 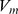 because of the electronic interaction, via 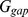. Therefore, the AP morphology changes as we have described above and shown in Figs. 2(a)–(f); to explain the results in this figure, we have to examine the behaviors of all the ionic currents when the myocyte is coupled to a fibroblast. For the ensuing discussion we consider a representative value of 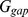, namely, 8.0 nS, and study the variation of the ionic currents as we change 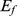 for an MF composite. In particular, we examine the time-dependence of the myocyte ionic currents 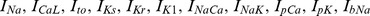, and 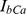, which are plotted in Fig. 4 for a fibroblast coupled with a myocyte, with 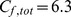 pF, 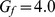 nS, 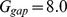 nS and 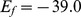 mV (blue squares) and 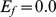 mV (red triangles); the full black curves with circles show the ionic currents for an uncoupled myocyte. We observe that, as we vary 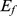, the 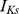 and 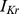 currents change substantially (see Figs. 4(d) and (e)). As a result, APD increases with increasing 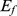, as shown in Fig. 2(a). Furthermore, as we increase 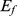, 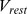 increases as shown in Fig. 2(b); the amount of elevation of 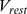 depends on (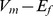). By examining the contributions of all ionic currents (see Fig. 4) to their values in the resting state of the AP (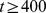 ms), we conclude that 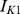 changes most significantly compared to other ionic currents. Therefore, we find the 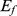-dependence of 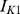 shown in Fig. 4(f). As we have noted above for an isolated myocyte, 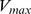 and 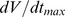 depend principally on 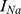; therefore, we examine 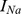 to understand the variations of 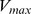 and 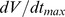 as functions of 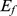 for an MF composite. We find that the magnitude of 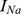 decreases as 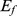 increases (Fig. 4(a)), so 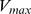 and 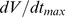 decrease as 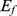 increases (as shown in Figs. 2(c) and (d)). Similarly, we look at 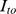 to understand the dependence of 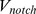 on 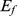; and we examine 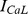 and 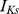 for the 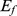-dependence of 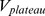. Figure 4(c) shows that 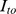 decreases when 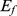 increases, therefore, 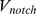 increases as a function of 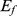 (as shown in Fig. 2(e)). Figures 4(b) and (d) show 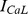 and 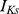, respectively; the former decreases and the latter increases as 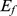 increases; however, the effect of 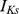 dominates that of 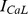 so 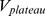 increases as 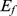 increases as shown in Fig. 2(f).

It has been noted in Refs. [37], [55], [56], that a myocyte cell can display autorhythmicity when it is coupled with fibroblasts; in particular, Ref. [55] shows that the cycle length of autorhythmicity activation depends on 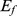 and 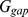. We find that 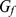 and 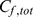 play a less important role than 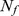, 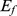, and 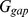 in determining whether such autorhythmicity is obtained. We show that the myocyte can behave like (a) an excitable, (b) autorhthymic, or (c) oscillatory cell depending on the value of 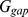 as shown and discussed in detail in Fig. S4 in Material S1.

**Figure 5:**
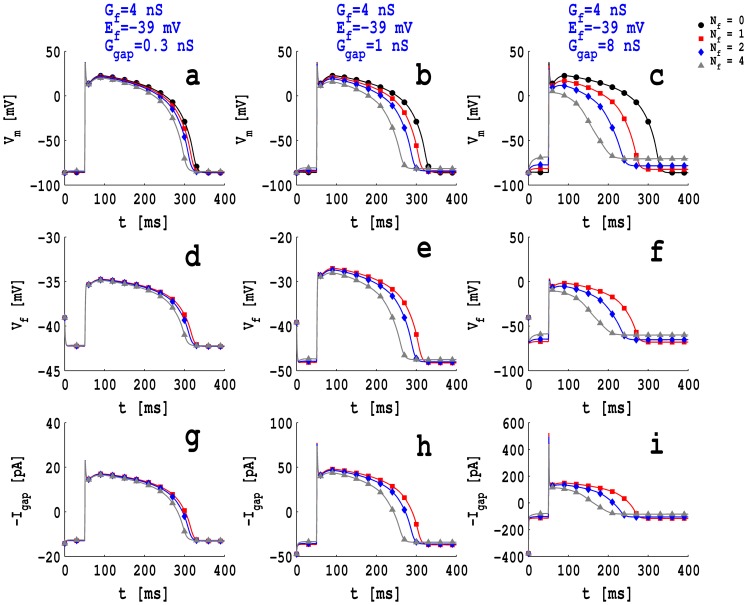
Plots versus 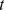 of 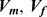, and 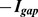: for 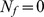, i.e., no fibroblasts (black circles), 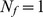 (red squares), 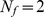 (blue diamonds), and 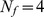 (gray triangles) for the illustrative parameter values 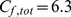 pF, 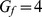 nS, 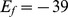 mV, and a low value of 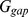, namely, 0.3 nS (plots in the left panel (a), (d), and (g)), an intermediate value of 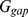, namely, 1.0 nS (plots in the middle panel(b),(e), and (h)), and a high value of 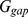, namely, 8.0 nS (plots in the right panel (c), (f), and (i)).

The number of fibroblasts 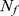 that are coupled to a myocyte in our MF composite affect significantly the response of the MF composite to external electrical stimuli as has been shown in detail in Refs. [9], [57]. In Fig. 5, we show illustrative plots versus 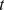 of 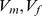, and 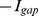 for our MF composite with 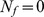, i.e., no fibroblasts (black circles), 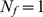 (red squares), 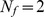 (blue diamonds), and 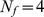 (gray triangles) for the parameter values 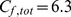 pF, 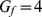 nS, 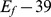 mV. For a low value of 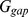, namely, 0.3 nS (plots in the left column), we see that the resting potential of the coupled myocyte is elevated slightly relative to the case 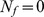; and the APD decreases from 280 ms, for 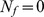, to 260 ms when 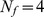 (Fig.5(a)); the dependence of 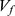 and 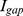 on 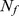 is illustrated in Figs.5(d) and (g). This dependence of 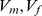, and 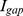 increases as we can see from the plots in the middle column, Figs.5(b), (e), and (h), for an intermediate value of 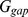, namely, 1 nS, and from the plots in the right column, Figs.5(c), (f), and (i), for an high value of 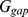, namely, 8 nS. In the former case (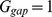 nS) 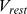 rises from −84.6 mV to −83.2 mV and the APD decreases from 280 ms to 240 ms as we go from 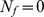 to 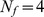 (Fig.5(b)); in the latter case (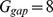 nS) 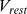 rises from −84.6 mV to −74.4 mV and the APD decreases from 280 ms to 150 ms as we go from 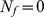 to 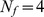 (Fig.5(c)).

In Table 2, we show the change of the APD, 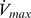, and 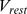 for an MF composite with respect to their uncoupled values when 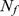 identical fibroblasts are coupled with a myocyte with high value of both 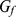 (4 nS) and 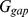 (8 nS). We measure 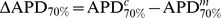, where 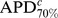 and 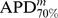 are, respectively, APD of an MF composite and isolated myocyte at 70% repolarization. Similarly, 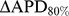 and 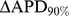 are, respectively, the change of the APD of MF composite with respect to the myocyte APD at 80% and 90% repolarization of AP. We do not present here the results of composites with 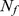 fibroblasts, which show AP automatically in the absence of external stimulus. The change of 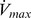, 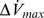, is measured by subtracting 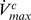 of an MF composite form an isolated myocyte 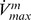. Similarly, the change of 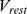, 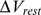, is measured by subtracting 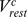 of an MF composite form an isolated myocyte 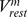. The analog of Table 2 for low values of 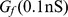 and 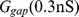, is given in Table S1 in the Material S1.

**Table 2:** The values of 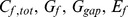, and 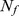 for a single MF composite and the changes in the AP morphology, relative to that of an uncoupled myocyte.

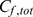 (pA)	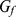 (ns)	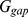 (ns)	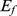 (mV)	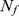	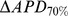 (ms)	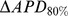 (ms)	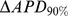 (ms)	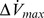 (mV/ms)	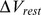 (mV)
6.3	4	8	−9	1	4.62	6.72	12.30	−91.89	7.12
6.3	4	8	−9	2	40.90	50.40	–	−151.68	14.99
6.3	4	8	−19	1	−16.80	−15.16	−11.02	−80.64	6.04
6.3	4	8	−19	2	−16.92	−11.02	–	−137.32	12.60
6.3	4	8	−29	1	−36.12	−34.84	−31.74	−68.40	4.97
6.3	4	8	−29	2	−58.00	−54.02	–	−122.88	10.33
6.3	4	8	−29	3	−64.56	−51.84	–	−270.10	15.91
6.3	4	8	−39	1	−53.62	−52.64	−50.32	−54.41	3.90
6.3	4	8	−39	2	−89.68	−86.92	−75.54	−103.87	8.21
6.3	4	8	−39	3	−112.60	−106.02	–	−135.49	12.21
6.3	4	8	−39	4	−126.26	−106.18	–	−270.10	16.35
6.3	4	8	−49	1	−69.52	−68.82	−67.10	−40.90	2.86
6.3	4	8	−49	2	−115.22	−113.30	−107.18	−82.25	6.18
6.3	4	8	−49	3	−146.06	−142.18	–	−112.77	9.10
6.3	4	8	−49	4	−173.62	−166.16	–	−134.10	11.77
6.3	4	8	−49	5	−195.72	−179.94	–	−149.25	14.30
6.3	4	8	−49	6	−205.32	−144.06	–	−162.44	16.72
6.3	4	8	−49	7	−214.12	–	–	−178.78	18.96

We concentrate on the APD, 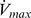, and 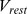 and list the changes, indicated by 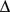, in these parameters. 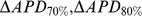, and 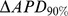 denote, respectively, the changes in the APD at 70%, 80%, and 90% repolarization. Note that here we have high values (see text) for both 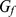 (4 nS) and 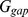 (8 nS).

### 2 Wave Dynamics in a 2D Simulation Domain with MF Composites

We move now to a systematic study of the propagation of electrical waves of activation in a 2D simulation domain with MF composites that are coupled via the types of intercellular and gap-junctional couplings described in the section on “Model and Methods”. We begin with an examination of plane-wave propagation through such a simulation domain and study the dependence of the conduction velocity 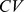 on the gap-junctional coupling 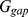 for zero-, one-, and two-sided couplings. We then study spiral-wave propagation in this domain. Finally, we investigate spiral-wave propagation through an inhomogeneous, square simulation domain in which most of the domain consists of myocytes, but a small region comprises a square MF composite.

In most of our numerical simulations of 2D MF composite domains, we choose the following representative values: the total cellular capacitance 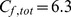 pF; the fibroblast conductance 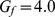 nS; the resting membrane potential of fibroblast 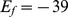 mV; and 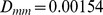 cm^2^/ms [15], which yields, in the absence of fibroblasts, the maximum value for the plane-wave conduction velocity 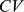, namely, −68.3 cm/s [17]; here 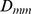 is the diffusion constant of the myocyte layer, and it is given by the relation, 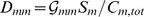, where 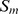 is the surface area of the myocyte. For the the gap-junctional conductance we explore values in the experimental range (see section on “Introduction”), 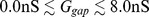; the remaining intercellular conductances lie in the following ranges: 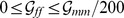 and 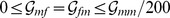. In some of our studies, we vary 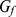 and 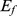; e.g., when we study spiral wave dynamics in the autorhymicity regime, we use 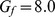 nS and 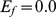 mV.

#### Spiral waves in homogeneous domains

As we have noted in the section on “Introduction”, both experimental and computational studies [13], [14], [26] have shown that 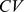 behaves nonmonotonically as a function of the number of fibroblasts 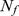 in an MF composite. However, to the best of our knowledge, no simulation has examined in detail the dependence of 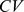 on 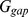; therefore, we examine this dependence for the zero-, single-, and double-sided couplings described in the section on “Model and Methods”.

**Figure 6:**
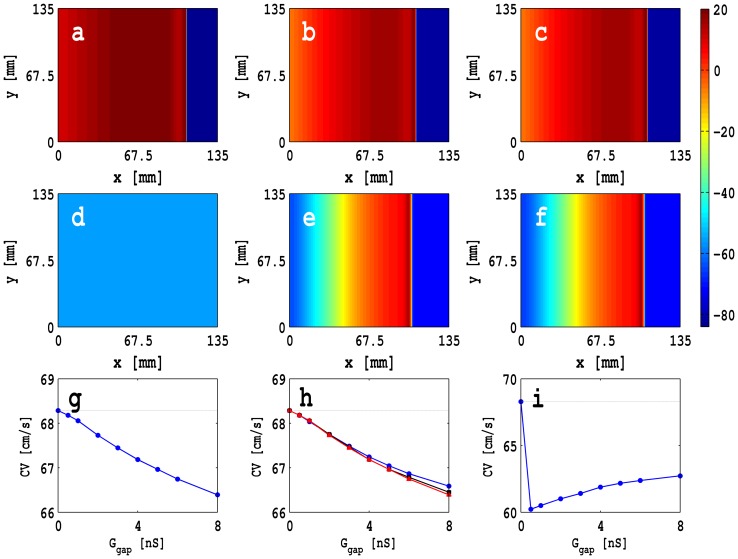
Plane waves shown via pseudocolor plots of 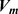 at 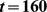 ms in a 2D square simulation domain of side 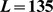 mm: for (a) the control case with only myocytes, (b) *zero-sided coupling*, (c) *single-sided coupling* with 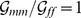, (d) *double-sided coupling* with 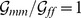, 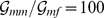, and 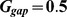 nS, (e) *double-sided coupling* with 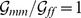, 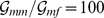, and 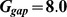 nS, and (f) *double-sided coupling* with 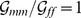, 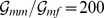, and 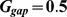 nS (Video S1 illustrates the spatiotemporal evolution of these plane waves). Plots of 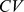 versus 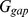 for (g) zero-sided coupling, (h) single-sided coupling with 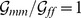 (blue circles), 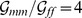 (black squares), and 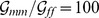 (red triangles), and (i) double-sided coupling with 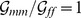 and 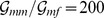. For double-sided coupling, conduction failure can occur for low and intermediate values of 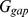, e.g., 0.5 nS and 2.0 nS, as shown in (d).

We measure 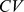 for a plane wave by stimulating the left boundary of the simulation domain with a current pulse of amplitude 150 pA/pF for 3 ms. This leads to the formation of a plane wave that then propagates through the conduction domain as shown in Fig. 6. For such a wave we can determine 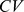 as described, e.g., in Refs. [16], [17]. In our *in silico* experiments, we observe that 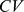 decreases monotonically, as a function of 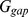, for zero- and single-sided couplings; but 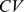 is a nonmonotonic function of 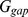 in the case of double-sided coupling. These behaviors of 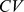, as a function of 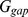, can be explained qualitatively by examining the dependence of the rate of depolarization 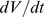 on 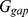 for an isolated MF composite. As we increase 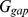, 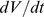 decreases because of the additional electrical load of the fibroblast on the myocyte; the fibroblast acts as current sink and, therefore, the flux carried by the wave front decreases and 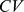 decreases. However, for *double-sided coupling*, 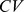 decreases initially and then increases as a function of 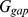 because of the cross-coupling terms 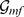 and 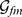. Such behaviors have been seen in earlier numerical studies, with passive or active fibroblast in models [13], [14] that are similar to, but not the same as, our mathematical model, and in cell cultures [13], [24]–[26].

We show plane waves in Figs. 6 (a)–(f) via pseudocolor plots of 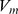 at 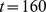 ms in a 2D square simulation domain of side 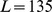 mm for (a) the control case with only myocytes, (b) *zero-sided coupling*, (c) *single-sided coupling* with 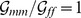, (d) *double-sided coupling* with 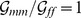, 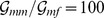, and 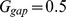 nS, (e) *double-sided coupling* with 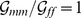, 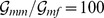, and 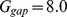 nS, and (f) *double-sided coupling* with 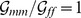, 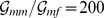, and 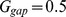 nS. Video S1 illustrates the spatiotemporal evolution of these plane waves. Figures 6 (g)–(h) show plots of 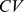 versus 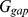 for (g) zero-sided coupling, (h) single-sided coupling with 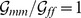 (blue circles), 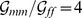 (black squares), and 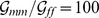 (red triangles), and (i) double-sided coupling with 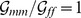 and 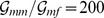. For double-sided coupling, conduction failure can occur for low and intermediate values of 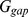, e.g., 0.5 nS and 2.0 nS, as shown in Fig. 6 (d); however, no conduction occurs in this range if 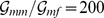. Such conduction failure is responsible for anchoring a spiral wave, at a localized MF composite, as we discuss in the next section where we describe our studies with inhomogenieties. We do not observe any conduction failure in the cases of zero- and one-sided coupling.

Two methods are often used to initiate spiral waves in simulations [15], [16], [58], [59] and in experiments [58], [60], namely, (1) the S1–S2 cross-field protocol and (2) the S1–S2 parallel-field protocol. In the cross-field method, a super-threshold stimulus S2 is applied at the boundary that is perpendicular to the boundary along which the S1 stimulus is given; in the parallel-field method, S2 is applied parallel to the refractory tail of the S1 stimulus, but not over the entire length of the domain. We use the parallel-field protocol to initiate a spiral wave in our homogeneous, square simulation domain with myocytes as follows. Initially we set the diffusion constant 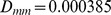 cm^2^/ms; this is a quarter of its original value, which is 0.00154 cm^2^/ms. We then inject a plane wave into the domain via an S1 stimulus of strength 150 pA/pF for 3 ms at the left boundary. After 560 ms we apply an S2 stimulus of strength 450 pA/pF for 3 ms just behind the *refractory tail* of the plane wave initiated by the S1 stimulus; in our simulation domain the S2 stimulus is applied over the region 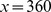 and 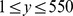. We reset 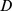 to its original value after 882 ms. This procedure yields a fully developed spiral wave at 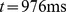 (see Fig. S5 in Material S1 for a pseudocolor plot of the transmembrane potential for this spiral wave); we use the values of 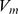, the gating variables, the intracellular ion concentrations for this spiral wave as the initial condition for our subsequent studies. By decreasing 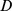 for this small interval of time we are able to reduce 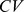 (because 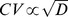) and, thereby, the wave length 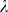; if we do not reduce 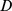 for this period, it is difficult to trap the hook of the proto spiral our simulation domain, whose side 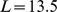 cm.

We use the fully developed spiral wave of Fig. S5 in Material S1 in (c) as an initial condition for the myocytes in our 2D simulation domain with MF composites; for the fibroblasts 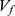 is set equal to 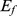 at this initial time.

**Figure 7:**
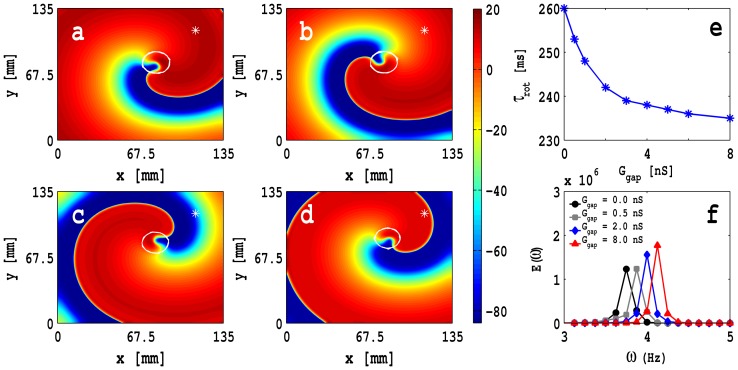
Pseudocolour plots of 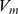 with zero-sided couplings: 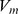
 in a square simulation domain of side 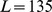 mm, at time 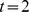 s with 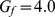 nS, 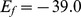 mV, with 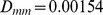 cm^2^/s, and (a) 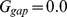 nS (control case, i.e., only myocytes), (b) 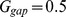 nS (low coupling), (c) 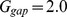 nS (intermediate coupling), and (d) 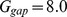 nS (high coupling); the white solid lines show the trajectory of the spiral tip for 2 s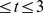 s. Video S2 illustrates the spatiotemporal evolution of these spiral waves. (e) Plot of the rotation period 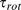 of the spiral wave versus 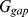. (f) Plots of the power spectra 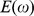 of the time series of 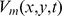 recorded from the representative point (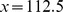 mm, 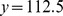 mm), which is indicated by an asterisk in (a)-(d), for 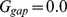 nS (black circles), (b) 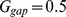 nS (gray squares), (c) 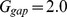 nS (blue diamonds), and (d) 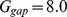 nS (red triangles); for these power spectra we use time series with 400000 points.

We begin by studying the dependence on 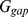 of spiral-wave dynamics in our 2D MF-composite simulation domain with zero-sided coupling. In Fig. 7 we show pseudocolour plots of 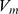, in a square simulation domain of side 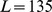 mm, at time 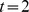 s with 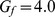 nS, 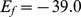 mV, and (a) 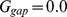 nS (control case, i.e., only myocytes), (b) 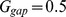 nS (low coupling), (c) 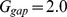 nS (intermediate coupling), and (d) 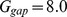 nS (high coupling); the white solid lines show the trajectory of the spiral tip for 2 s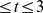 s. Video S2 illustrates the spatiotemporal evolution of these spiral waves. The plot in Fig. 7 (e) of the rotation period 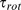 of the spiral wave versus 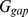 shows that 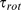 decreases as 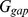 increases. In Fig. 7 (f) we show the power spectra 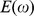 of the time series of 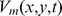 recorded from the representative point 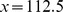 mm,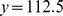 mm, which is indicated by an asterisk in Figs. 7 (a)-(d), for 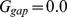 nS (black circles), (b) 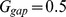 nS (gray squares), (c) 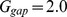 nS (blue diamonds), and (d) 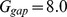 nS (red triangles); for these power spectra we use time series with 400000 points; the discrete lines in these power spectra show that, over these time scales, we have periodic temporal evolution that is a characteristic signature of a single, rotating spiral wave. The position of the fundamental peak in these power spectra moves to high frequencies as we increase 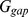 in a manner that is consistent with the decrease of 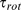 in (e).

The rotation period 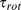, for zero-sided coupling, decreases as 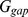 increases. We can understand this qualitatively by looking at the APD of an MF composite and examining the propagation properties of plane waves that we have discussed above. Recall that, as 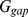 increases, the myocyte APD for a myocyte in an MF composite decreases for low values of 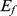 (e.g., 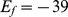 mV, as shown by red triangles in Fig. 2 (a)). The APD is roughly equal to the refractory time of a plane wave in a 1D or 2D domain [61], therefore, the refractory period of a plane wave in 2D decreases as 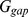 increases; and we have checked explicitly that it decreases as 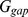 increases by applying an additional stimulus at the wave back of the propagating plane wave. The rotation period 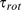 of a spiral wave is related to the refractory period of a plane wave, although the curvature plays an additional role in the spiral-wave case [62], [63]. Therefore, spiral waves rotate faster as 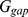 is increased.

**Figure 8:**
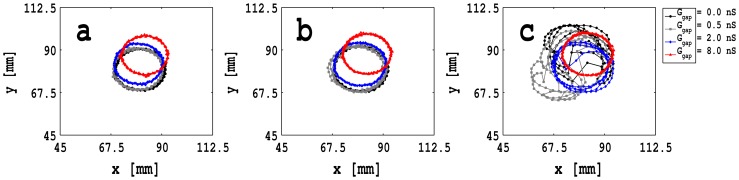
Plots of spiral-wave-tip trajectories with zero-sided couplings: Spiral-wave tip trajectories for the time intervals (a) 2 s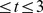 s, (b) 4 s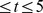 s, and (c) 6 s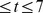 s, with zero-sided coupling, and for the control case with 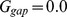 ns (black circles), 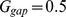 ns (gray squares), 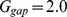 ns (blue diamonds), and 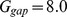 ns (red triangles), and all other parameter values as in Fig. 7. Videos S2, S3, and S4 illustrate the spatiotemporal evolution of these spiral-tip trajectories.

The white solid lines in Figs. 7 (a)–(d) show the trajectories of the spiral tips for 2 s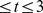 s. If we monitor these trajectories for longer durations of time, we obtain the tip trajectories shown in Figs. 8 (a), (b), and (c), respectively, for the time intervals 2 s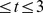 s, 4 s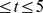 s, and 6 s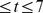 s for the case of zero-sided coupling, for the control case with 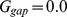 ns (black circles), 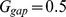 ns (gray squares), 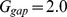 ns (blue diamonds), and 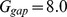 ns (red triangles), and all other parameter values as in Fig. 7. Note that in Figs. 8 (a) and (b), i.e., for 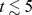 s, these trajectories are very nearly circular for all the values of 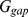 we consider. However, for 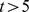 s, the tip trajectories can form 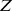-type curves as shown in Fig. 8 (c) for the control case with 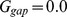 ns (black circles), 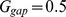 ns (gray squares), and 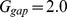 ns (blue diamonds); the trajectory for 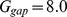 ns (red triangles) continues to be circular. Videos S2, S3, and S4 illustrate the spatiotemporal evolution of these spiral-tip trajectories. The stability of the spiral core increases as we increase 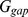 because the interaction of the wave back of the preceding arm of the spiral wave and the wave front of the following arm decreases with increasing 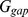.

We also study spiral-wave dynamics in both autorhythmic and oscillatory regimes as shown and discussed in detail in Fig. S6 in Material S1.

**Figure 9:**
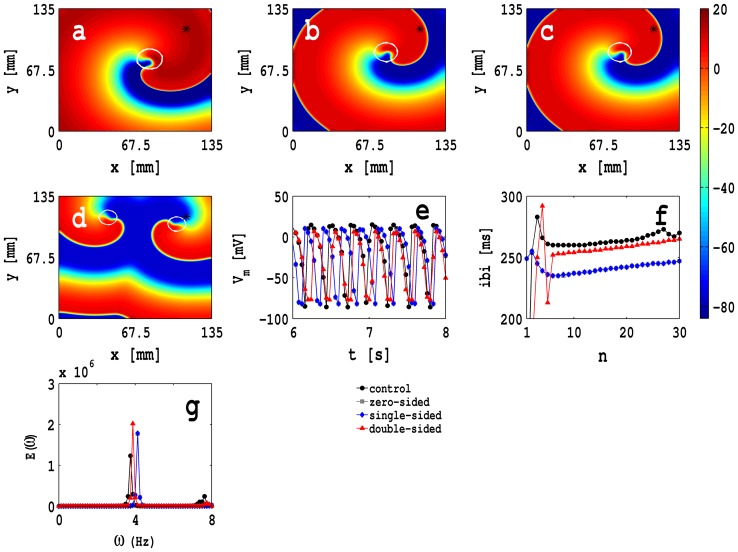
Spiral waves with zero-, one-, and two-sided couplings: Pseudocolor plots of 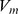 at time 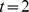 s in a simulation domain with 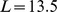 cm, an MF composite at every site, with a myocyte M coupled via 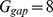 nS with one fibroblast F (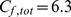 pF, 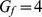 nS, and 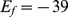 mV) for (a) control case with only myocytes and no fibroblasts, (b) zero-sided coupling, (c) single-sided coupling with 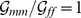, and (d) double-sided coupling with 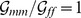 and 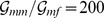; the white solid lines in plots (a), (b), (c), and (d) show the trajectories of the spiral tip for 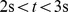. Video S5 illustrates the spatiotemporal evolution of these spiral waves. (e) Time series data for 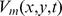 are recorded at the point 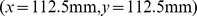, shown by an asterisk, for 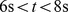. (f) Plot of the inter-beat interval (ibi) versus the beat number 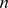, and (g) the power spectrum 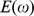 (of 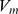) versus the frequency 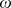 for the control case (black circles), zero-sided coupling (gray squares), single-sided coupling (blue diamonds), and double-sided coupling (red triangles); these plots of ibi and 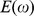 are obtained from a time series of 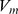, with 400000 data points separated by 0.02 ms.

In Fig. 9 we show spiral waves via pseudocolor plots of 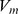 at time 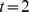 s; here, at each site, we have an MF composite with a myocyte M coupled with one fibroblast F for which 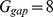 nS, 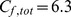 pF, 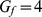 nS, and 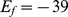 mV. Figure 9(a) shows the control case in which there are only myocytes and no fibroblasts; Fig. 9(b) shows the spiral wave for the case of zero-sided coupling; Fig. 9(c) gives the spiral wave for single-sided coupling with 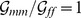; and Fig. 9(d) portrays this wave when we have double-sided coupling with 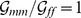 and 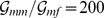; the white solid lines in these plots show the trajectories of the spiral tip for 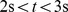. Video S5 illustrates the spatiotemporal evolution of these spiral waves. In Fig. 9(d), we show the time series of 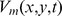, in the interval 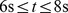, obtained from a representative point, shown by asterisks in Figs. 9(a-d), namely, (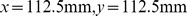), for the control case (black circles), zero-sided coupling (gray squares), single-sided coupling (blue diamonds), and double-sided coupling (red triangles); Figs 9(e) and (f) contain, respectively, plots of the inter-beat interval (ibi) versus the beat number 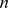 and the power spectrum 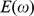 (of 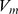) versus the frequency 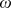 for the control case (black circles), zero-sided coupling (gray squares), single-sided coupling (blue diamonds), and double-sided coupling (red triangles); these plots of ibi and 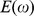 are obtained from a time series with 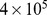 data points separated by 0.02 ms and recorded from the representative point 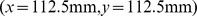 that is indicated by a * in these pseudocolor plots. With double-sided couplings, we have seen the formation of two spirals (see Fig. 9(d)), given the spiral-initialization procedure that we use. From the animations in Video S5, the time series of 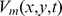, the ibi, and the power spectra in Fig. 9, we conclude that the spiral rotates faster for single-sided coupling than for zero-sided coupling; the spiral rotation rate in the double-sided case lies between these rates for zero- and one-sided couplings. Such fast and slow rotation rates of spiral waves, for these three types of couplings, can be understood by analyzing the AP morphology of a single MF composite and the 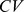 of plane waves, with these three types of couplings. In general, our studies have shown that, if the fibroblasts in the MF composites act as current sources (current sinks), then the rate of rotation of the spiral wave increases (decreases).

#### Spiral waves in inhomogeneous domains

We now examine the effects of fibroblast inhomogeneities on spiral-wave dynamics in our mathematical model; outside the region of the inhomogeneity we use the TNNP model for cardiac tissue; inside the inhomogeneity we use the 2D MF composite domain that we have used in our studies above.

We model an inhomogeneity in our 2D simulation domain by incorporating a small square patch of side 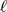; this patch is an MF composite domain; the remaining part of the simulation domain contains only myocytes that are coupled via 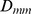. Again, we focus on three different types of couplings in the MF-composite domain, namely, zero-, single-, and double-sided couplings between myocytes and fibroblasts; and we choose to the following, representative fibroblast parameters: 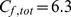 pF, 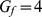 nS, and 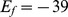 mV. In most of our studies, we use 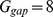 nS.

**Figure 10:**
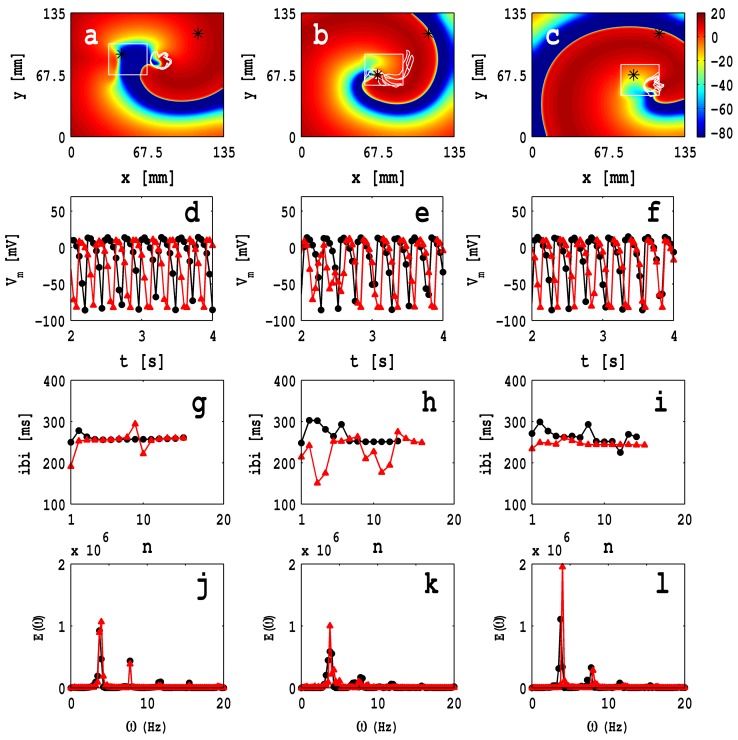
Pseudocolor plots of the transmembrane potential of the myocyte 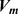 at time, 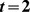 s, in the presence of a square shape MF composite inhomogeneity, of side 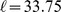 mm, for the case of zero-sided coupling; the bottom-left corner of the inhomogeneity is fixed at (a) 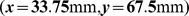 , (b) 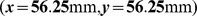 , and (c) 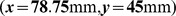 ; the white solid lines in these figures show the spiral-tip trajectories in the time interval 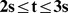 and the local time series data are recorded from points that are shown by asterisks. Video S6 illustrates the spatiotemporal evolution of these spiral waves. The plots in (d)-(f) show the time series for 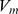, in the interval 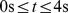, which are obtained from a point outside (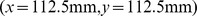 for all cases) and inside the fibroblast inhomogeneity (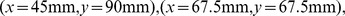 and 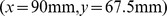 for (a), (b), and (c), respectively), represented by black circles and red triangles, respectively; (g), (h), and (i) show plots of the inter-beat intervals (ibis) versus the beat number 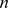 for the time series of 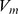 mentioned above; each one of these time series contain 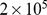 data points; the power spectra 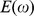, which follow from these time series, are given in (j), (k), and (l).

In Figs. 10 (a), (b), and (c) we show pseudocolor plots of 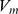 at time, 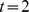 s, in the presence of a square fibroblast inhomogeneity, of side 33.75 mm, for the case of zero-sided coupling, and the lower-left-hand corner of the inhomogeneity at, respectively, 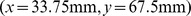, 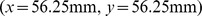 and 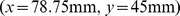, respectively; the white solid lines in these figures show the spiral-tip trajectories, for 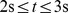. We also obtain time series for 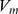 from a point outside the inhomogeneity (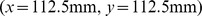 for all cases) and a point inside it (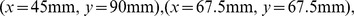 and 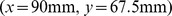 for Figs. 10 (a), (b), and (c), respectively). Data from the points outside and inside the fibroblast inhomogeneity are represented, respectively, by black circles and red triangles in Figs. 10 (d), (e), and (f). Figures 10 (g), (h), and (i) show plots of the inter-beat intervals (ibis) versus the beat number 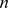 for the time series of 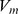 mentioned above; each one of these time series contain 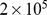 data points; the power spectra 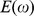, which follow from these time series, are given in Figs. 10 (j), (k), and (l).

The white solid lines in Figs. 10 (a)–(c) show the trajectories of the spiral tips for 2 s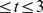 s; note that these trajectories depend sensitively on the position of the MF composite inhomogeneity; furthermore, the dynamics of the wave, inside and outside this inhomogeneity, are different (cf., our previous studies with ionic inhomogeneities [16], [17]). Figure 10 (a) shows that, in the presence of an MF-composite inhomogeneity, the tip trajectory deviates from one with a circular core; the corresponding plots of the ibis and power spectra, Figs. 10 (g) and (j), respectively, illustrate that the temporal evolution of the spiral is periodic inside and outside of the inhomogeneous domain. However, for the inhomogeneity of Fig. 10 (b), we observe non-periodic and periodic temporal evolutions, respectively, inside and outside the MF inhomogeneity as can be surmised from the ibi and power-spectra plots in Figs. 10 (h) and (k); the tip has a 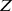-type trajectory, extends over a length 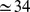 mm, and it meanders both inside and outside the inhomogeneity. For the inhomogeneity of Fig. 10 (c), we obtain periodic and non-periodic temporal evolutions, respectively, inside and outside the MF-composite inhomogeneity (see Figs. 10 (i) and (l) for the ibi and power spectra); here the tip trajectory has a linear extent 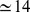 mm and it is restricted, predominantly, inside the inhomogeneity. The Video S6 has four animations that show superimpositions of pseudocolor plots of 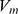 and the spiral-tip trajectories for 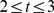 s for a control myocyte layer with no inhomogeneities and the simulation domains for Figs. 10 (a)–(c). If we use one-sided coupling with 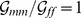, we find that our results are similar to those we have depicted in Fig. 10 for zero-sided coupling, as we show in Fig. S7 in Material S1. In general, the heterogeneity of cardiac tissue causes a drift of the spiral wave towards regions in which a spiral wave has a long period of rotation [64]–[66]. In our studies, the period of a spiral wave decreases slightly inside the fibroblast heterogeneity; and the final position of the spiral depends on the location of the heterogeneity.

**Figure 11:**
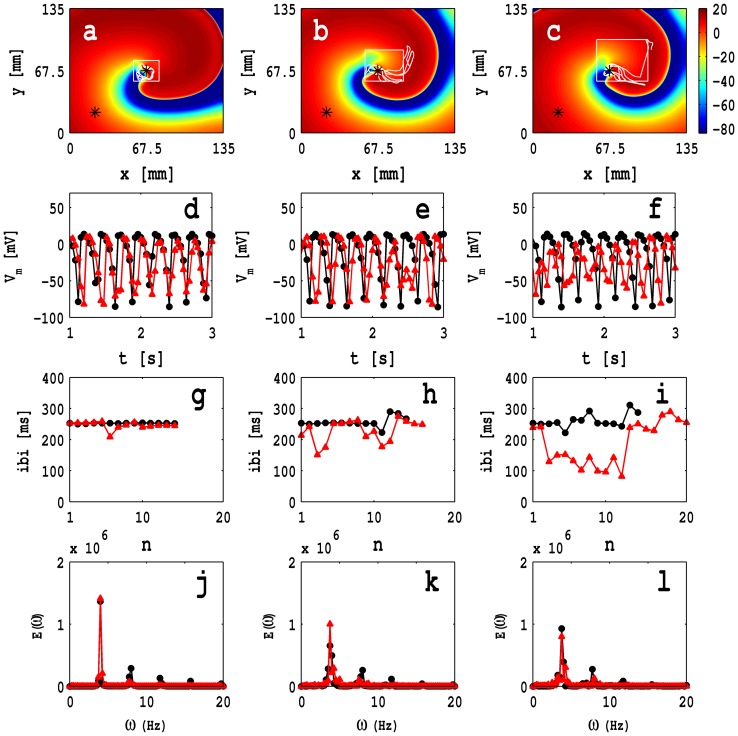
Pseudocolor plots of 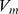 with MF-composite inhomogeneities: Pseudocolor plots of the transmembrane potential of the myocyte 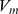 at time, 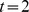 s, in the presence of three square MF-composite inhomogeneities with sides (a) 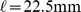, (b) 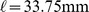, and (c) 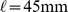, for the case of zero-sided coupling; the bottom-left corner of these squares is fixed at 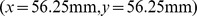; the white solid lines in these figures show the spiral-tip trajectories in the time interval 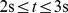 and the local time series data are recorded from points that are shown by asterisks. Video S7 illustrates the spatiotemporal evolution of these spiral waves. The plots in (d)-(f) show the time series for 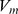, in the interval 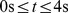, which are obtained from the point outside the inhomogeneity 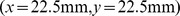 and a point inside it 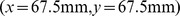, represented by black filled circles and red filled triangles, respectively; (g), (h), and (i) show plots of the inter-beat intervals (ibis) versus the beat number 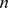 for the time series of 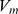 mentioned above; each one of these time series contain 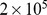 data points; the power spectra 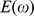, which follow from these time series, are given in (j), (k), and (l).

Next we investigate the dependence of spiral-wave dynamics on the size of the MF-composite inhomogeneity. Figures 11 (a), (b), and (c) show pseudocolor plots, at time 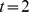 s, of 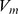 for three representative square inhomogeneities, with sides 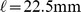, 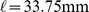, and 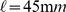; the lower-left-hand corner of these squares is fixed at 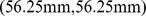. The white, solid lines in these figures show the spiral-tip trajectories for 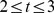 s. The spiral-tip trajectory, shown in Fig. 11(a), is a closed, but not circular, path that is confined inside the inhomogeneity; as the size of the inhomogeneity grows, this tip trajectory also grows in size and parts of it lie outside the inhomogeneity, as shown, e.g., in Fig. 11(b) and (c); in the latter two cases, the tip trajectories are not closed and their linear extent is comparable to the length of the side of the inhomogeneity. We also obtain time series for 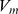 from a point outside the inhomogeneity 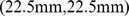 and a point inside it 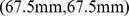; these points are indicated, respectively, by black and white asterisks in Figs. 11 (a), (b), and (c); and data from the points outside and inside the fibroblast inhomogeneity are represented, respectively, by black circles and red triangles in Figs. 11 (d)-(l). In Figs. 11 (d), (e), and (f) we give the time series of 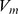; Figs. 11 (g), (h), and (i) show plots of the inter-beat intervals (ibis) versus the beat number 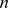 for the time series of 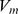 mentioned above (these contain 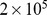 data points); the power spectra 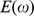, which follow from these time series, are given in Figs. 11 (j), (k), and (l). The Video S7 has four panels that show superimpositions of pseudocolor plots of 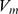 and the spiral-tip trajectories for 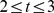 s for a control myocyte layer with no inhomogeneities (top left panel) and the simulation domains for Figs. 11 (a), (b), and (c) (top right, bottom left, and bottom right panels, respectively). We observe rich varieties of spiral-wave dynamics, both inside and outside of the inhomogeneity. The precise spatiotemporal evolution of the spiral waves depends on the size of the inhomogeneity; for a careful investigation of this size dependence, we must keep one point of the inhomogeneity, say its left bottom corner, fixed, as in Figs. 11(a), (b), and (c), where we find that the system moves from periodic to non-periodic temporal evolution as the size of the inhomogeneity increases; this conclusion follows from the ibi plots in Figs. 11(g)-(i) and the power spectra in Figs. 11(j)-(l). Note, furthermore, that the spiral-wave rotation period 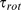 decreases as the size of inhomogeneity increases; this is consistent with Fig. 9 (f), which shows that the ibi for a homogeneous myocyte layer is greater than the ibi of a homogeneous MF-composite layer.

**Figure 12:**
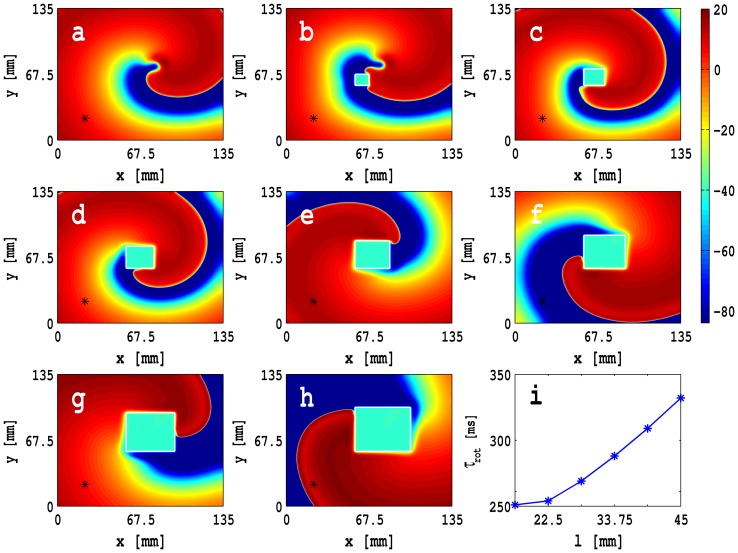
Pseudocolor plots of the transmembrane potential of the myocyte 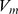 at time, 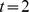 s, when a square MF-composite inhomogeneity, with 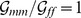 and 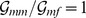, is placed with its lower-left corner at 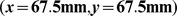; the square has a side of length (a) 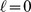, i.e., no inhomogeneity, (b) 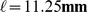, (c) 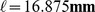, (d) 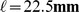, (e) 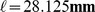, (f) 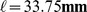, (g) 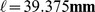, and (h) 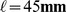. Video S8 illustrates the spatiotemporal evolution of these spiral waves for (a), (d), (f) and (h). The smallest MF-composite inhomogeneity that can anchor a spiral wave has 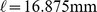; (i) shows a plot of the rotation period 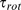 of such an anchored spiral wave.

We turn now to a study of an MF-composite inhomogeneity with double-sided coupling. We have shown that, in a homogeneous, 2D simulation domain with such MF composites, the occurrence of conduction block depends on the value of 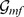; e.g., we have observed that conduction failure occurs if 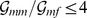 and 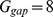 nS, but it does not occur if 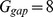 nS and 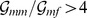; in the latter case, 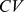 depends on the ratio 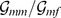. Therefore, we choose the following four representative values for 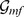 in our MF-composite inhomogeneity studies: 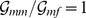, 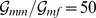, 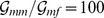, and 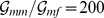. We begin with 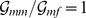 for which conduction failure occurs for all physical values 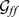 and 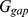 in the homogeneous case. In Figs. 12 (a)–(h), we show pseudocolor plots of 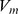 at time 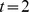 ms, when a square MF-composite inhomogeneity, with 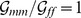 and 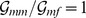, is placed with its lower-left corner at 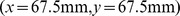; the square has a side of length 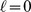, i.e., no inhomogeneity (Fig. 12(a)), 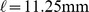 (Fig. 12(b)), 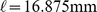 (Fig. 12(c)), 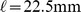 (Fig. 12(d)), 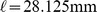 (Fig. 12(e)), 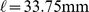 (Fig. 12(f)), 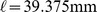 (Fig. 12(g)), and 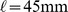 (Fig. 12(h)). The smallest MF-composite inhomogeneity that can anchor a spiral wave has 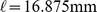. The Video S8 has four panels that show the spatiotemporal evolution of pseudocolor plots of 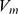 for 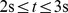 for a control myocyte layer with no inhomogeneities (top left panel) and the simulation domains for Figs. 12 (d), (f), and (h) (top right, bottom left, and bottom right panels, respectively). Figure 12(i), a plot of the rotation period 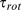, of such an anchored spiral wave, versus 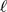, shows how 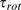 increases with 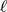; such an increase has also been seen for a conduction inhomogeneity [67].

If the value of 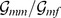 is such that conduction failure occurs in a homogeneous, MF-composite simulation domain, then the MF-composite inhomogeneity behaves somewhat like a conduction inhomogeneity inasmuch as the spiral wave does not enter significantly into the region of the inhomogeneity. We discuss in detail in Material S1 (Figs. S8) how far a spiral wave penetrates into an MF-composite inhomogeneity. We find that, depending on the values of parameters, the MF-composite inhomogeneity can act like a conduction inhomogeneity, or, the spiral wave can penetrate the region of the inhomogeneity marginally, i.e., the MF-composite inhomogeneity can act like an ionic inhomogeneity [16].

**Figure 13:**
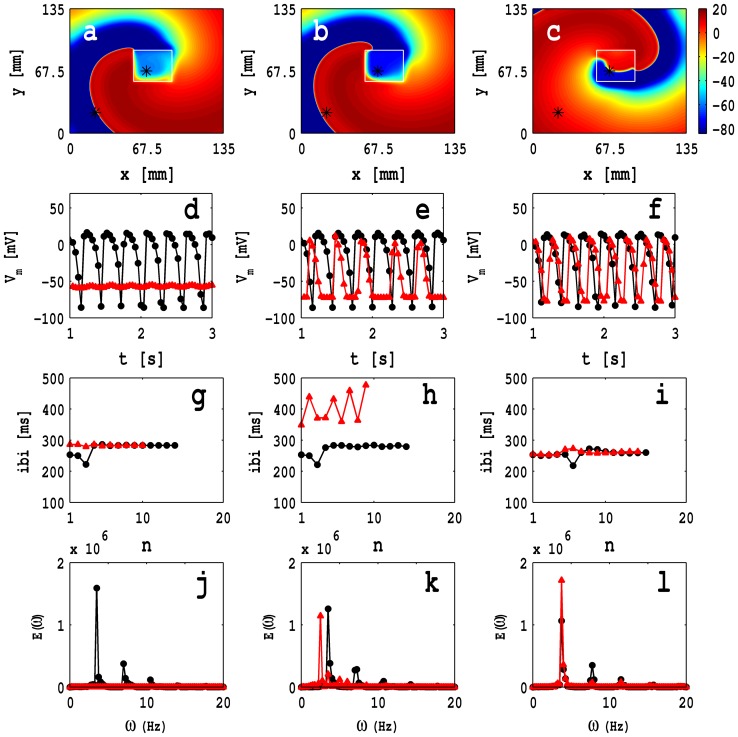
Pseudocolor plots of the transmembrane potential of the myocyte 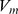, at time 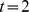 s, in the presence of a square, MF-composite inhomogeneity, of side 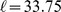 mm and with its lower-left-hand corner placed at 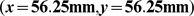 for the case of doubled-sided coupling with 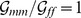 and 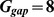 nS: (a) 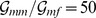, (b) 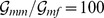, and (c) 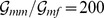. Video S9 illustrates the spatiotemporal evolution of these spiral waves. The time series for 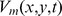, with 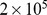, from a point outside the inhomogeneity (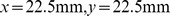) and a point inside it (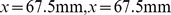), both of which are depicted by asterisks in (a)-(c), are plotted in (d), (e), and (f) for 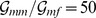, 100, and 200, respectively (data from the points outside and inside the inhomogeneity are represented, respectively, by black circles and red triangles); (g), (h), and (i) show the corresponding plots of the ibi versus the beat number 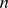; and the associated power spectra 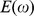 are depicted in (j), (k), and (l).

In our plane-wave studies in 2D homogeneous simulation domains with double-sided coupling, we have noted that the propagation speed 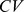 and the wave length 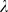 depend 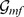. Therefore, we now carry out a study of the interaction of spiral waves with an MF-composite inhomogeneity for different values of 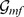. We have seen above that an MF-composite inhomogeneity behaves somewhat like a conduction inhomogeneity if 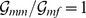. In Figs. 13 (a), (b), and (c) we show, for 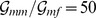, 100, and 200, respectively, pseudocolor plots of 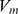, at time 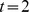 s, in the presence of a square, MF-composite inhomogeneity, of side 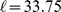 mm and with its lower-left-hand corner placed at 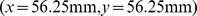 for the case of doubled-sided coupling with 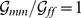 and 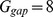 nS. We also obtain time series for 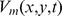 from a point outside the inhomogeneity (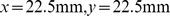) and a point inside it (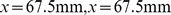), both of which are depicted by asterisks in Figs. 13 (a)-(c). These time series, with 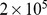 data points each, are plotted in Figs. 13 (d), (e), and (f) for 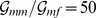, 100, and 200, respectively (data from the points outside and inside the inhomogeneity are represented, respectively, by black circles and red triangles); Figs. 13 (g), (h), and (i) show the corresponding plots of the ibi versus the beat number 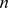; and the associated power spectra 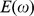 are depicted in Figs. 13 (j), (k), and (l). The Video S9 has four panels that show the spatiotemporal evolution of pseudocolor plots of 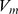 and the spiral-tip trajectories for 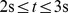 for a control myocyte layer with no inhomogeneities (top left panel) and, in addition, the simulation domains of Figs. 13 (a)-(c) (top right, bottom left, and bottom right panels). We observe rich spiral-wave dynamics, which can be different inside and outside of the MF-composite inhomogeneity, as in the cases with zero- and single-sided couplings. The degree to which the spiral-wave penetrates inside the inhomogeneity depends on the value of 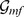, as we can see from the time-series plots of Figs. 13(d)-(f). We examine the interaction of spiral waves with an MF-composite inhomogeneity for different values of 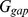 in detail in Material S1 (Fig. S9). The Video S10 has four panels that show the spatiotemporal evolution of pseudocolor plots of 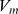 and the spiral-tip trajectories for 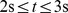 for a control myocyte layer with no inhomogeneities (top left panel) and, in addition, the simulation domains of Figs. S9(a)-(c) in Material S1 (top right, bottom left, and bottom right panels). Here too we obtain a rich variety of spiral-wave behaviors inside and outside of the MF-composite inhomogeneity, as in the cases with zero- and single-sided couplings.

### 3 Control of Spiral-wave Turbulence in the Presence of MF-composite Inhomogeneities

One of the goals of our extensive studies of various types of heterogeneities in mathematical models for cardiac tissue [16], [17], [68], [69] has been to understand their effects on spiral-wave dynamics and thus develop effective, low-amplitude control techniques for the elimination of single, rotating spiral waves or spatiotemporally chaotic multiple spiral waves of electrical activation in mathematical models for cardiac tissue. In these earlier studies [16], [17], [68], [69] we have considered conduction or ionic inhomogeneities; here we have extended such studies to mathematical models in which we allow for the MF-composite inhomogeneities that we have described above.

In this subsection we investigate the elimination of spiral-wave turbulence in the presence of MF-composite inhomogeneities. We use the control scheme of Sinha, *et al.*, [19]; this eliminates spiral waves by the application of a current pulse on a mesh, which we describe below. We have found in our earlier studies [16]–[19] that such a mesh-based control scheme is effective even when the simulation domain has conduction or ionic inhomogeneities; by contrast, control schemes, which use electrical stimuli at a point [70], [71], work well in homogeneous simulation domains but do not eliminate spiral-wave turbulence in domains with inhomogeneities.

In our mesh-based control scheme in a 2D simulation domain with an MF-composite inhomogeneity, we apply a current pulse of amplitude 30 pA/pF for 400 ms over a mesh that divides our square simulation domain, of side 135 mm, into 16 square cells of side 33.75 mm each. The application of this pulse makes the region, which is covered by the mesh, refractory and, therefore, effectively imposes Neumann boundary conditions for any cell bounded by this mesh. Thus, spiral waves that lie inside the cell are absorbed at the mesh that bounds it and, eventually, spiral-wave turbulence is eliminated from the whole simulation domain.

**Figure 14:**
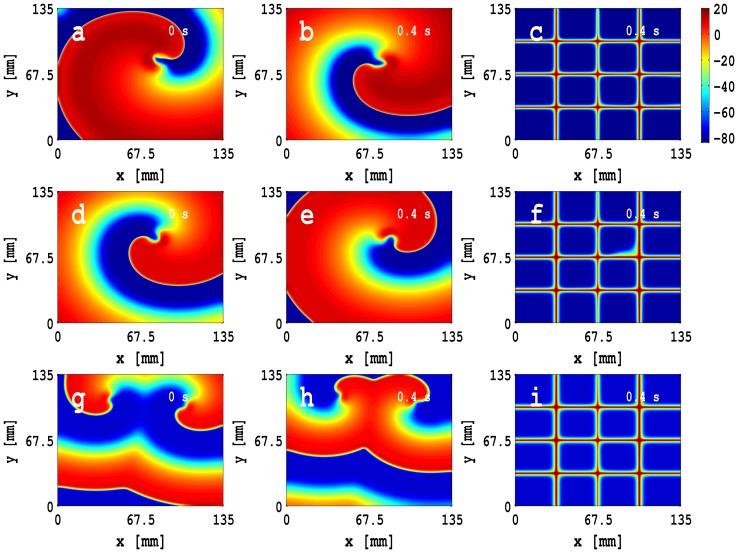
Spiral-wave control in our 2D M-F composite model, by the application of a control pulse of amplitude 30 pA/pF for 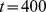 ms over a square mesh with each block of side 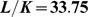 mm, i.e., the simulation domain is divided into 4^2^ square blocks. Plots in (a), (d) and (g) are the initial conditions of 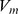, i.e., 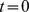 ms, for the control case, i.e., 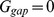 nS, zero sided coupling with 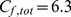 pF, 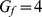 nS, 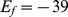 mV and 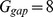 nS, and double-sided coupling with the same fibroblasts parameters as in (d) and with 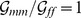 and 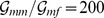, respectively; (b), (e) and (h) show the pseudocolor plots of 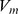 at 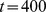 ms in the absence of any control pulse. However, the spiral-wave is suppressed by an application of the control pulse as shown (c), (f) and (i) at time 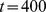 ms. The Video S11, which comprises six animations of pseudocolor plots of 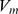, shows the spatiotemporal evolution of these spiral waves for these cases, with and without control pulses.

We begin with a discussion of the control of spiral waves, in a 2D, MF-composite simulation domain, by the application of a current pulse on the square mesh described above. In Fig. 14 (a) we show a pseudocolor plot of 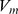 at time 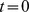 ms, for the control case with 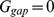 nS; we give pseudocolor plots of 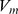, at 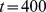 ms, and in the absence and presence of the control pulse in Figs. 14 (b) and (c), respectively. Figures 14 (d), (e) and (f), are the analogs of Figs. 14 (a), (b), and (c), respectively, for zero-sided coupling with 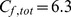 pF, 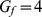 nS, 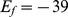 mV, and 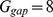 nS. Figures 14 (g), (h), and (i) are the analogs of Figs. 14 (a), (b), and (c), respectively, for two-sided coupling with 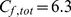 pF, 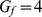 nS, 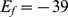 mV, 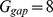 nS, 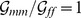, and 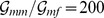. From the pseudocolor plots of 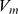 in Figs. 14 (c), (f), and (i) we see that our mesh-based, spiral-control scheme succeeds in eliminating spiral-wave turbulence in less than 400 ms in a 2D, MF-composite simulation domain with zero- and two-sided couplings; we have obtained similar results with one-sided coupling too. The Video S11, which comprises six animations of pseudocolor plots of 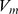, shows the spatiotemporal evolution of the spiral waves for these cases, with and without control pulses.

**Figure 15:**
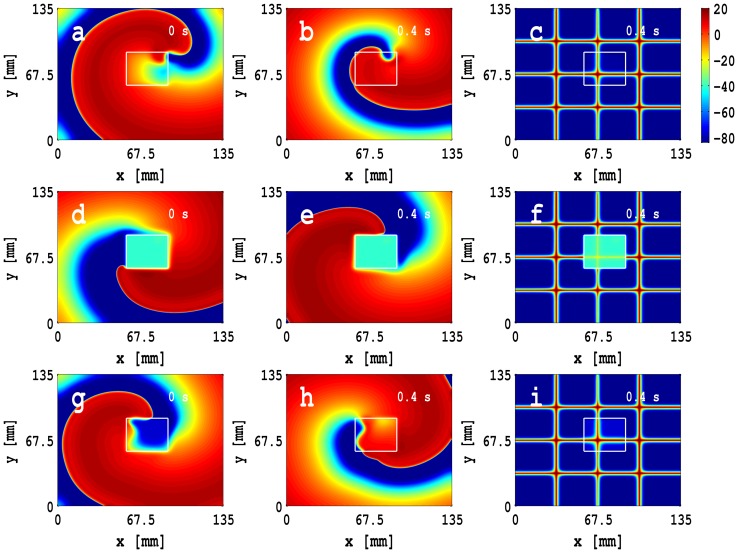
Spiral-wave control in the 2D Fibroblast model in the presence of a square shape MF composite inhomogeneity of size 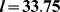 mm whose bottom-left corner is placed at (56.25 mm, 56.25 mm). We apply a control pulse of amplitude 30 pA/pF for 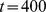 ms over a square mesh with each block of size 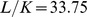 mm, i.e., the simulation domain is divided into 4^2^ square blocks. Plots in Figs. (a), (b) and (c) are the initial conditions of 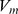, i.e., 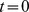 ms, for zero sided coupling with 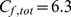 pF, 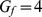 nS, 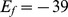 mV and 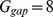 nS, double sided coupling with same fibroblasts parameters as in (a) with 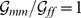 and 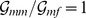, and double sided coupling with same fibroblasts parameters as in (a) with 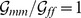 and 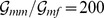, respectively. Figures (b), (e) and (h) are the time evolution of 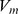 corresponds to Figs. (a), (d), (g), respectively, at 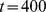 ms in absence of any control pulse. However, the spiral-wave is suppressed an application of control pulse as shown in Figs. (c), (f) and (i) at time 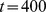 ms. For the spatiotemporal evolution of these spiral waves see Video S12.

We now study spiral-wave control in a 2D simulation domain with myocytes and a square MF-composite inhomogeneity with side 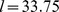 mm whose bottom-left corner is placed at 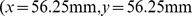. Again, we apply a control pulse of amplitude 30 pA/pF for 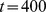 ms over a square mesh with cells whose sides are of length 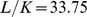 mm, i.e., the simulation domain is divided into 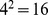 square blocks. We consider the following three cases: (A) zero-sided coupling with 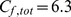 pF, 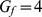 nS, 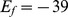 mV and 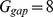 nS; (B) double-sided coupling with the same fibroblasts parameters as in case (A) and with 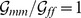 and 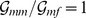; and (C) double-sided coupling with the same fibroblasts parameters as in case (A) and with 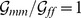 and 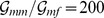. Figures 15 (a), (d), and (g) show pseudocolor plots of 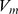 at 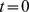 for cases (A), (B), and (C), respectively. Figures 15 (b), (e), and (h) show their analogs for 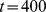 ms when no control pulse is applied. Figures 15 (c), (f), and (i), which show pseudocolor plots of 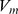 at 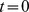 for cases (A), (B), and (C), respectively, at 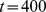 ms when we apply a control pulse, illustrate how our control scheme is effective in suppressing spiral-wave turbulence in the presence of an MF-composite inhomogeneity. For the spatiotemporal evolution of these spiral waves see Video S12.

## Discussion and Conclusion

We have carried out detailed numerical studies of an MF composite by modelling human ventricular myocyte cells, as in Ref. [15], and fibroblasts as passive RC circuits, as in Refs. [9], [12]. The passive nature of these fibroblasts makes them behave as inexcitable cells and, therefore, they act either as current sources or sinks, when they are coupled with myocytes. We have investigated the responses of such MF composites to external electrical stimuli by varying systematically the total cellular capacitance 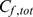 of fibroblasts and their membrane conductance 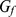, resting membrane potential 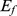, gap-junctional coupling (with myocytes) 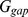, and the number 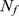 of fibroblasts coupled to a myocyte over a wide range of biophysically relevant values [3], [5], [46], [49], [50]. We find that the myocyte APD increases as we increase 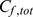 in the fibroblast; but it does not significantly affect the shape of the AP. An increase in 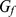 prolongs the APD, if the resting potential of the fibroblast is high (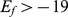 mV), but it shortens the APD if 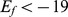 mV; and 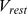 is elevated substantially as 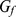 is increased. We focus mainly on the dependence of the AP morphology on 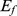 and 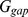, because the values of these two parameters span a wide range in experiments [3], [5], [49], [50]. We find that the APD increases with 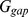 (Fig. 2), if 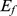 is high, but it decreases, as 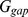 increases, if 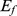 is low. The maximum upstroke velocity, 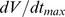, and the maximum value of the myocyte transmembrane potential, 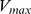, decrease as we increase 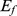 (Fig. 2), with a fixed value of 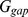, whereas the notch of the AP, 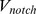, the maximum of the plateau, 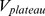, and the resting membrane potential all increase as we increase 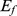. Furthermore, we have shown that 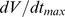, 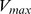, 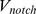, and 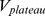 decrease, but 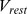 increases, as we increase 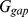 (Fig. 2), with a fixed value of 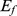. We have carried out simulations to check the dependence of the myocyte AP on the number 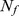 of fibroblasts, which are coupled to a myocyte. We have observed that (a) the APD and 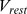 decrease and increase, respectively, as 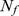 increases and (b) this increase of 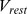 depolarizes the membrane potential so that, eventually, 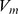 crosses the threshold value for the generation of an AP, and, therefore, the MF composite cell begins to show either autorhythmic or oscillatory behaviors [57].

Fibroblasts are found to be much smaller than myocytes in experimental studies [8], [72]; thus, in cell-culture experiments, more than one fibroblast can be deposited per myocyte in the cell culture. Furthermore, the sizes of fibroblasts can vary in such experiments; this depends on the preparation technique and circumstances; and the fibroblast size decides the maximum number of fibroblasts that can attach to a myocyte. Indeed, a wide range of values has been used for the total cellular capacitance 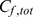 in various computer models [9], [11], [12] because these models assume that 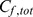 is related to the size of the fibroblast. Hence, in our model studies, for a given size of fibroblast, i.e., a fixed value of 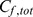, the complex, network-type interaction can occur via 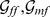, and 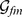, depending on the number of fibroblasts 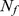 coupled to a myocyte in an MF composite. Therefore, we use 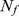 fibroblasts per a myocyte site in an MF composite to study the wave dynamics; each of these fibroblasts are coupled to a myocyte via 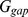. We think of this collection of 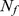 fibroblasts as a single cluster that interacts via 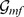 and 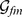, with its neighboring myocytes; we assume that the number of fibroblast clusters is exactly equal to the number of myocyte cells. We assume that, at the level of a first approximation, the fibroblasts in a cluster interact with each other only to the extent that they form cluster. When the size of the fibroblast cluster is much smaller than the size of a myocyte, then the couplings 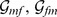, and 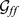 are irrelevant and we should only use a zero-sided model.

Earlier computational studies [9]–[12], which we have discussed in the “Introduction” section, have not investigated the dependence of the AP morphology, as we do, on the parameters 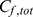, 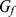, 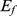, 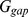, and 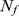; most of these earlier studies have focused on the dependence of the AP on 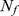, principally, and 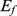, to some extent. Our detailed studies of the dependence of the AP morphology on 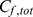, 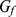, 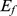, 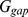, and 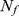 are designed to help experimentalists in the growth of different tissue layers with myocytes and fibroblasts and thus uncover the contribution of fibroblasts to the mechanisms of ventricular fibrillation. For example, in our simulation, we have found that 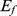 has the potential to alter the APD and, therefore, it can play a crucial role in spiral-wave dynamics.

We have performed extensive numerical simulations in two-dimensional (2D) simulation domains, both homogeneous and inhomogeneous, which contain myocytes or MF composites with zero-, one-, or two-sided couplings between myocytes and fibroblasts. We have found that, for zero- and one-sided couplings, the plane-wave conduction velocity 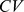 decreases as 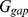 increases, but, with two-sided couplings, 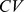 first decreases very rapidly and then rises slowly as 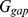 increases; furthermore, we have found that conduction failure can occur in the double-sided case if 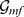 and 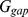 are low (Fig. 6). Such behaviors have been seen in earlier numerical studies, with passive or active fibroblast in models [13], [14] that are similar to, but not the same as, our mathematical model, and in cell cultures [13], [24]–[26]. In our studies with zero-sided coupling, we have found that (a) the rotation period 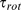 of a spiral wave decreases as we increase 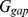 (Fig. 7), which we have explained qualitatively, (b) the higher the value of 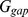, the more compact and closed is the trajectory of the spiral tip (this compactness prevents a single spiral from splitting into multiple spirals [73]–[75]), and (c) in the parameter range in which autorhythmicity occurs, the spiral wave rotates periodically, for low frequencies (Figs. S6 in Material S1).

Our studies of spiral-wave dynamics in a homogeneous simulation domain with MF composites has shown that the spiral rotates faster for single-sided coupling than for zero-sided coupling. The spiral rotation rate in the double-sided case lies between these rates for zero- and one-sided couplings. We have presented a qualitative explanation of such fast and slow rotation rates of spiral waves by analyzing the AP morphology of a single MF composite and the plane-wave 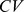 with these three types of couplings. In general, our studies have shown that, if the fibroblasts in the MF composites act as current sources (current sinks), then the rate of rotation of the spiral wave increases (decreases).

Our studies of spiral-wave dynamics in the presence of localized, MF-composite inhomogeneities have shown that they may block spiral-wave propagation like conduction inhomogeneities [16], [28], [30], [68], [69], [76]; such behavior is the consequence of conduction failure, which we have observed in our plane-wave studies in homogeneous domains. However, in some cases the spiral wave can enter the region with the MF-composite inhomogeneity and yield rich spiral-wave dynamics, which can be different inside and outside of the inhomogeneity, as happens, e.g., when we have ionic inhomogeneities [16], [31], [69]. We have found that zero- and single-sided couplings have the potential to behave like ionic inhomogeneities; but double-sided couplings have the potential to behave either like conduction- or ionic-type inhomogeneities (depending on the value of 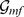). Furthermore, spiral-wave dynamics, with all these three types of couplings, depends sensitively on the position and size of our MF composite inhomogeneities [16], [17].

Our model has some limitations: it does not have any mechanosensitive currents [77], either for the myocytes or the fibroblast. These types of ionic currents have been obtained in experimental studies of fibroblasts in the sino-atrial node (SAN) and atria of certain mammals [3], [46], [48], [77], [78]. These mechanosensitive currents may affect spiral-wave propagation if there is electro-mechanical feedback [79], [80]; our model excludes such electro-mechanical feedback. The purely electrical approach, which we adopt, has also been used by several other groups (see, e.g., Refs. [19], [76], [81]–[86]) with the understanding that the mechanical system basically follows the electrical activation at the level of a first approximation (see, e.g., Ref. [87]). Furthermore, our model is based on passive fibroblasts rather than active fibroblast; there are several reasons to use passive fibroblast rather than active ones, the main reason being that, so far, no experiments have identified the presence of ionic currents in human, ventricular fibroblasts; and the presence of active fibroblast in human, ventricular fibroblasts continues to be a matter of debate. The expression of ionic currents in fibroblasts have been observed in animal species, either in the region of the SAN or the right atrium. In spite of these limitations of our model, our work provides the most comprehensive study, attempted so far, of (a) the response of MF composites to external electrical stimulation and (b) the propagation of spiral waves in MF-composite 2D simulation domains, which are designed to model cell-culture experiments of the sort presented in Refs. [13], [26]. We do not attempt here to study diffuse fibrosis; this lies beyond the scope of this paper; it has been addressed in other simulation studies [40], [57], [88]–[90]. Our studies have been designed specifically to uncover the role of fibroblasts in spiral-wave dynamics in the the absence of other tissue heterogeneities; once the underlying contributions of fibroblasts to spiral-wave dynamics have been revealed, we can incorporate tissue anisotropy and diffuse fibrosis as, e.g., in Ref. [57].

One of our goals is to test our low-amplitude control scheme in our 2D MF-composite model; we have focused here on a monodomain model, because we use low-amplitude control pulses rather than high-amplitude ones; the latter may require a bidomain model. Furthermore, some studies [91] have shown that there are no significant qualitative differences between bidomain and monodomain models, so we expect that our principal qualitative result will continue to hold even when such models are considered; this will have to be checked explicitly by subsequent studies.

## Supporting Information

Material S1 Supplementary Material
(PDF)

Video S1Plane-wave propagation in our 2D, homogeneous myocyte-fibroblast (MF) model for different cases. (a) the control case, i.e., with only myocytes; (b) *zero-sided coupling*; (c) *single-sided coupling* with 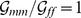; (d) *double-sided coupling* with 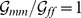, 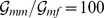, and 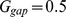 nS; (e) *double-sided coupling* with 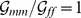, 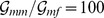, and 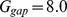 nS; and (f) *double-sided coupling* with 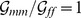, 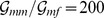, and 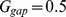 nS. The time evolution of pseudocolor plots of the myocyte transmembrane potential 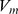 is shown for 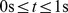; we use 10 frames per second (fps); in real time each frame is separated from the succeeding frame by 8 ms.(MPG)

Video S2Spiral-wave dynamics in our 2D homogeneous myocyte-fibroblast model with zero-sided coupling. (a) 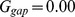 nS (control case, i.e., with only myocytes); (b) 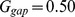 nS (low coupling); (c) 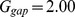 nS (intermediate coupling); (d) 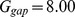 nS (high coupling). Here spiral-tip trajectories, for 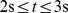, are shown by the white lines; we use 10 frames per second (fps); in real time each frame is separated from the succeeding frame by 8 ms.(MPG)

Video S3Spiral-wave dynamics in our 2D homogeneous myocyte-fibroblast model with zero-sided coupling. (a) 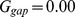 nS (control case, i.e., with only myocytes); (b) 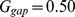 nS (low coupling); (c) 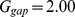 nS (intermediate coupling); (d) 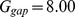 nS (high coupling). Here spiral-tip trajectories, for 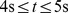, are shown by the white lines; we use 10 frames per second (fps); in real time each frame is separated from the succeeding frame by 8 ms.(MPG)

Video S4Spiral-wave dynamics in our 2D homogeneous myocyte-fibroblast model with zero-sided coupling. (a) 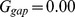 nS (control case, i.e., with only myocytes); (b) 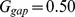 nS (low coupling); (c) 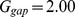 nS (intermediate coupling); (d) 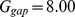 nS (high coupling). Here spiral-tip trajectories, for 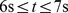, are shown by the white lines; we use 10 frames per second (fps); in real time each frame is separated from the succeeding frame by 8 ms.(MPG)

Video S5Spiral-wave dynamics in our 2D homogeneous myocyte-fibroblast model with gap-junctional conductance, 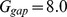 nS. (a) control case; (b) zero-sided coupling; (c) single-sided coupling; (d) double-sided coupling. Here spiral-tip trajectories, for 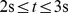, are shown by the white lines; we use 10 frames per second (fps); in real time each frame is separated from the succeeding frame by 8 ms.(MPG)

Video S6Spiral-wave dynamics in our 2D myocyte-fibroblast model in the presence of a square fibroblast inhomogeneity, of side 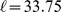 mm, for the case of zero-sided, and the lower left-hand corner of the inhomogeneity at different positions. (a) control case; (b) (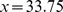 mm, 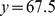 mm); (c) (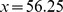 mm, 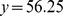 mm); (d) (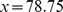 mm, 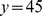 mm). Here spiral-tip trajectories, for 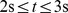, are shown by the white lines; we use 10 frames per second (fps); in real time each frame is separated from the succeeding frame by 8 ms.(MPG)

Video S7Spiral-wave dynamics in our 2D myocyte-fibroblast model in the presence of a square fibroblast inhomogeneity of side 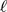 for the case of zero-sided with the lower-left-hand corner of the inhomogeneity fixed at (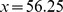 mm, 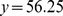 mm). (a) control case; (b) 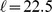 mm; (c) 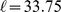 mm; (d) 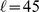 mm. Here spiral-tip trajectories, for 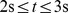, are shown by the white lines; we use 10 frames per second (fps); in real time each frame is separated from the succeeding frame by 8 ms.(MPG)

Video S8Spiral-wave dynamics in our 2D myocyte-fibroblast model with a double-sided coupling and a square fibroblast inhomogeneity with side 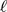 and 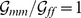 and 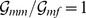, and the lower left-hand corner of the inhomogeneity fixed at (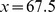 mm, 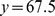 mm). (a) 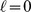, i.e., absence of inhomogeneity; (b) 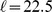 mm; (c) 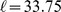 mm; (d) 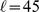 mm. Here the myocyte transmembrane potential time evolution is shown for 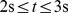; we use 10 frames per second (fps); in real time each frame is separated from the succeeding frame by 8 ms.(MPG)

Video S9Spiral-wave dynamics in our 2D myocyte-fibroblast model in the presence of a square, MF-composite inhomogeneity, of side 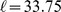 mm and with its lower-left-hand corner placed at (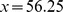 mm, 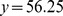 mm) for the case of doubled-sided coupling with 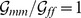 and 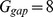 nS. (a) control case, i.e., with only myocytes; (b) 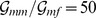; (c) 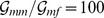; (d) 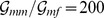. Here the myocyte transmembrane potential time evolution is shown for 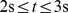; we use 10 frames per second (fps); in real time each frame is separated from the succeeding frame by 8 ms.(MPG)

Video S10Spiral-wave dynamics in our 2D myocyte-fibroblast model in the presence of a square, MF-composite inhomogeneity, of side 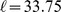 mm and with its lower-left-hand corner placed at (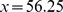 mm, 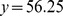 mm) for the case of doubled-sided coupling with 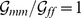 and 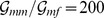. (a) control case, i.e., with only myocytes; (b) 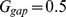 nS (low coupling); (c) 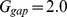 nS (intermediate coupling); (d) 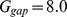 nS (high coupling). Here the myocyte transmembrane potential time evolution is shown for 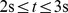; we use 10 frames per second (fps); in real time each frame is separated from the succeeding frame by 8 ms.(MPG)

Video S11Spiral-wave dynamics, without (top panel) and with (bottom panel) control pulses, in our 2D MF-composite model; we apply a control pulse of amplitude 30 pA/pF for 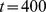 ms over a square mesh with each block of side 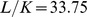 mm, i.e., the simulation domain is divided into 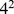 square blocks. (a) for the control case, i.e., 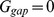 nS; (b) zero-sided couplings with 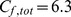 pF, 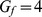 nS, 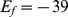 mV and 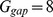 nS; and (c) double-sided couplings with the same fibroblasts parameters as in (b) and with 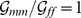 and 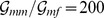. The animations in (d), (e), and (f) are the analogs of (a), (b), and (c), respectively, with control pulses. Here the spatiotemporal evolution of the myocyte transmembrane potential is shown for 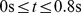; we use 10 frames per second (fps); in real time each frame is separated from the succeeding frame by 8 ms.(MPG)

Video S12Spiral-wave dynamics, without (top panel) and with (bottom panel) control pulses, in the 2D Fibroblast model in the presence of a square MF composite inhomogeneity of side 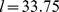 mm whose bottom-left corner is placed at (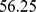 mm, 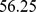 mm); we apply a control pulse of amplitude 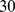 pA/pF for 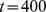 ms over a square mesh with each block of side 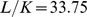 mm, i.e., the simulation domain is divided into 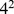 square blocks. (a) zero-sided coupling with 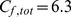 pF, 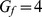 nS, 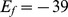 mV and 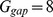 nS, (b) double-sided coupling, with the same fibroblasts parameters as in (a) with 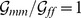 and 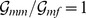, and (c) double-sided coupling with the same fibroblasts parameters as in (a) with 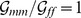 and 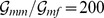. The animations in (d), (e), and (f) are the analogs of (a), (b), and (c), respectively, with control pulses. Here the spatiotemporal evolution of the myocyte transmembrane potential is shown for 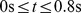; we use 10 frames per second (fps); in real time each frame is separated from the succeeding frame by 8 ms.(MPG)
