# Supplementary material for: Spiral-Wave Dynamics in a Mathematical Model of Human Ventricular Tissue with Myocytes and Fibroblasts
Source: PLoS One. 2013 Sep 4;8(9):e72950. doi: 10.1371/journal.pone.0072950 (PMC3762734; doi:10.1371/journal.pone.0072950)
Supplement: Material S1 — Supplementary Material (PDF) [file pone.0072950.s001.pdf]

Supplementary Material S1

Spiral-wave dynamics in a Mathematical Model of Human Venticular Tissue with Myocytes and Fibroblasts

Alok Ranjan Nayak[1], T.K. Shajahan[2], A.V. Panfilov[3], and Rahul Pandit[1]

1 Centre for Condensed Matter Theory,
Department of Physics, Indian Institute of Science,
Bangalore 560012, India.
2 Centre for Nonlinear Dynamics in Physiology and Medicine,
McGill University,
Montreal, Canada.
3 Department of Physics and Astronomy,
Gent University,
Krijgslaan 281, S9,
9000 Gent, Belgium.

Our principal results have been given in the main paper of this paper. We present additional results of our numerical simulations of (a) MF composites and (b) two-dimensional (2D) arrays of such units (see Section on "Model and Methods") in this Supplementary Material S1.

I. Results

a. A Myocyte-Fibroblast (MF) Composite

The ranges of parameters, which we use for our composite MF system, are consistent with those found in experimental studies and those used in earlier computational studies. For example, in a cell-culture experiment, Rook, *et al.* [1] have studied rat-heart fibroblasts and reported that the membrane resistance R_f, the fibroblast resting membrane potential E_f, and the gap-junctional conductance G_{gap}, lie, respectively, in the ranges $3-25$ $G\Omega$, -20 to -40 mV, and $0.3-8.0$ nS. Kohl *et al.* [2] have studied non-excitable cardiac, mechanosensitive fibroblasts from the region of the sinoatrial node in a rat heart. Their study, which uses both intact tissue and cell cultures, estimates that $R_f \simeq 1$ $G\Omega$, $E_f \simeq -15 \pm 10$ mV, and $G_{gap} \simeq 4-6$ nS for a well-coupled MF pair. In vitro studies, by Kiseleva, *et al.* [3], have examined rat mechanosensitive fibroblasts attached to the right atrium; they have found $E_f \simeq -22 \pm 1.9$ mV and $R_f \simeq 0.51 \pm 0.01$ $G\Omega$, for a control case, and $E_f \simeq -46.5 \pm 1.8$ mV and $R_f \simeq 3.8 \pm 0.03$ $G\Omega$, in the case of a large infarct caused by a myocardial infarction. In vitro studies by Kamkin, *et al.* [4] of non-excitable, mechanosensitive, cardiac fibroblasts from the atrium of a human heart have reported $E_f \simeq -15.9 \pm 2.1$ mV $R_f \simeq 4.1 \pm 0.1$ $G\Omega$. In vitro studies, by Kamkin *et al.* [5], of rat atrial fibroblasts attached to the sinoatrial node region yielded $E_f \simeq -22 \pm 2$ mV and $R_f \simeq 510 \pm 10$ $M\Omega$, for the control case, and for the case with myocardial infarction, and $E_f \simeq -41 \pm 3$ mV to $\simeq -28 \pm 3$ mV. Recent experiment, in culture, by Chilton *et al.* [6] have measured the cellular capacitance $C_{f,tot}$ of rat-ventricular fibroblasts by using a patch-clamp recording and found $C_{f,tot} \simeq 6.3 \pm 1.7$ pF; they have shown that the input resistance of fibroblasts $R_f \simeq 10.7 \pm 2.3$ $G\Omega$. Their measurements have shown that E_f depends on the inwardly rectifying K^+ current (Kir) and the potassium ion concentration $[K^+]_o$; e.g, when Kir is expressed, E_f is $\simeq -65 \pm 5$ mV and $\simeq -80 \pm 1.8$ mV for $[K^+]_o = 10$ mM and 5.4 mM, respectively. However, when Kir is absent, E_f is $\simeq -34 \pm 2$ mV. Furthermore, in culture, Shibukawa, *et al.* [7] have found, in patch-clamp recordings from rat-ventricular fibroblasts (active), that $C_{f,tot} \simeq 4.5 \pm 0.4$ pF, $E_f \simeq -58 \pm 3.9$ mV, $R_f \simeq 5.5 \pm 0.6$ $G\Omega$.

The computational studies of mathematical models for fibroblasts, discussed in the "Introduction" Section of the main paper, have also used a wide range of values for parameters for the cellular capacitance $C_{f,tot}$, the membrane conductance G_f, the fibroblast resting membrane potential E_f, and the gap-junctional coupling G_{gap} between myocyte and fibroblasts. For example, Xie, *et al.* [8] have used $C_{f,tot} = 25$ pF, $G_f = 0.1-4$ nS, $E_f = -50-0$ mV, and $G_{gap} = 0-20$ nS for an MF composite. The study of

Sachse, *et al.* [9] has used $C_{f,tot}$ = 4.5 pF, E_f = −58 mV, and G_{gap} = 0.1 − 100 nS for an MF composite with active fibroblasts. Jacquemet, *et al.* [10] have studied the MF composite with active fibroblasts by using $C_{f,tot}$ = 4.5 pF, E_f = −58 mV, and G_{gap} = 0.09 − 4.05 nS. MacCannell, *et al.* [11] have used $C_{f,tot}$ = 6 − 60 pF, E_f = −49.6 mV and G_{gap} = 1 − 3 nS for their studies of an active-fibroblast model. To investigate in detail the effect of fibroblasts on a myocyte, we use the following wide ranges of parameters (these encompass the ranges used in the experimental and computational studies mentioned above): $C_{f,tot}$ = 6 − 60 pF, G_f = 0.1 − 4 nS, E_f = −39 to 0 mV, and G_{gap} = 0.3 − 8.0 nS for our MF composites. However, to observe some special properties, such as autorhythmicity of MF composites, we vary the fibroblast parameters and gap-junctional conductances.

It has been noted in Refs. [12–14], that a myocyte cell can display autorhythmicity when it is coupled with fibroblasts; in particular, Ref. [12] shows that the cycle length of autorhythmicity activation depends on E_f and G_{gap}. We find that G_f and $C_{f,tot}$ play a less important role than N_f, E_f, and G_{gap} in determining whether such autorhythmicity is obtained. In Fig. S4 we give some illustrative plots for N_f = 1, E_f = 0mV, and G_f = 8nS that yield autorhytmicity; Fig. S4 (a) shows a plot of V_m versus time t; Fig. S4 (b) contains a plot of the frequency of autorhthymicity f versus G_{gap} for our MF composite; for more detailed studies of the dependence of such autorhythmicity on N_f and E_f we refer the reader to Ref. [15]. Figure S4 (b) shows that, for the range 0 nS $\lesssim G_{gap} \lesssim$ 16 nS, the myocyte behaves like an excitable cell, which produces one action potential when it is stimulated electrically; in the range 16 nS $\lesssim G_{gap} \lesssim$ 23 nS, the myocyte displays autorhthymicity and the cycle length λ_{cl}, the time difference between the upstrokes of two successive action potentials, decreases with increasing G_{gap}; for $G_{gap} \gtrsim$ 23nS, the myocyte displays oscillatory behavior. Such autorhythmic and oscillatory responses of an MF composite [15] can occur at lower values of G_{gap}, e.g., G_{gap} = 8 nS, if we increase N_f.

b. Wave dynamics in a 2D simulation domain with MF composites

For the case of zero-sided coupling, with E_f = 0 mV and G_f = 8 nS, Figs. S6(a)-(c) show, respectively, pseudocolor plots of the myocyte transmembrane potential V_m, at time t = 2 s, for low-frequency autorhythmicity (e.g., with G_{gap} = 17 nS), high-frequency autorhythmicity (e.g., with G_{gap} = 20 nS), and when the MF composite displays (cf. Fig. S4) oscillatory behavior (e.g., with G_{gap} = 23 nS). In Fig. S6(d), we show the time series of $V_m(x, y, t)$, in the interval 0 s $\le t \le$ 4 s, obtained from three representative points, shown by asterisks in Fig. S6(a), namely, (x = 22.5 mm, y = 22.5 mm) (black filled circles), (x = 67.5 mm, y = 67.5 mm) (blue filled diamonds), and (x = 112.5 mm, 112.5 mm) (red filled triangles); Fig. S6(g) shows the corresponding power spectra, which we calculate from these time series, each of which has 2×10^5 data points; each one of these power spectra has discrete, sharp peaks at a fundamental frequency and at its harmonics; the periodic nature of the time series and these peaks in the power spectra provide evidence for the temporally periodic motion of the spiral wave in the region of low-frequency autorhythmicity. The analogs of Figs. S6(d) and (g) are shown in Figs. S6(e) and (h) for the regime of high-frequency autorhythmicity; the time series of $V_m(x, y, t)$, in the interval 0 s $\le t \le$ 4 s, from (x = 22.5 mm, y = 22.5 mm) (black filled circles) and (x = 67.5 mm, y = 67.5 mm) (blue filled diamonds), show irregular behaviors and the corresponding power spectra show subsidiary peaks near the main peaks; however, the time series recorded from (x = 112.5 mm, 112.5 mm) (red filled triangles) shows periodic behavior and, consequently, the corresponding power spectrum has discrete, strong peaks. The analogs of Figs. S6(d) and (g), for the oscillatory regime, are shown in Figs. S6(f) and (i).

If the value of $\mathcal{G}_{mm}/\mathcal{G}_{mf}$ is such that conduction failure occurs in a homogeneous, MF-composite simulation domain, then the MF-composite inhomogeneity behaves somewhat like a conduction inhomogeneity inasmuch as the spiral wave does not enter significantly into the region of the inhomogeneity. To check how far the wave penetrates into the MF-composite inhomogeneity, we show in Figs. S8 (a), (b), and (c) pseudocolor plots of V_m at times t = 2s, t = 6s, and t = 8s, respectively, when a square MF-composite inhomogeneity of side ℓ = 33.75mm, with $\mathcal{G}_{mm}/\mathcal{G}_{ff}$ = 1 and $\mathcal{G}_{mm}/\mathcal{G}_{mf}$ = 1 is placed with its bottom left corner at (x = 56.25 mm, y = 56.25 mm). Data for the time series of $V_m(x, y, t)$ are recorded at three points of the simulation domain, namely, (x = 90mm, y = 112.5mm), which lies outside the inhomogeneity, (x = 90mm, y = 90mm), at the top-right corner of the inhomogeneity, and (x = 90mm, y = 67.5mm), on the right-middle side of the inhomogeneity; these points are indicated by asterisks in Fig. S8 (c) and the data recorded from them are represented, respectively, by black circles, blue diamonds, and red triangles in Figs. S8 (d)-(f). Figure S8 (d) contains plots of the time series of V_m (each one of these time series contain 2×10^5 data points). Figure S8 (e) shows the corresponding plots of the inter-beat intervals (ibis) versus the beat number n; and the power spectra $E(\omega)$, which follow from the time series of V_m, are

given in Fig. S8 (f).

From the time series of V_m (Fig. S8 (d)), we see small-amplitude oscillations in V_m if the time series are obtained from points at the side and corner of the MF-composite inhomogeneity; however, if the point lies outside the inhomogeneity, this time series shows a periodic pattern of action potentials. These times series and the plots of the ibi (Fig. S8 (e)) show that the oscillations in V_m, from these three different points, are in phase; to this extent the MF-composite inhomogeneity acts like a conduction inhomogeneity [16]; however, the spiral wave does penetrate the region of the inhomogeneity marginally, so, in this sense, the MF-composite inhomogeneity acts like an ionic inhomogeneity [16].

We turn now to an examination of the interaction of spiral waves with an MF-composite inhomogeneity for different values of G_{gap}. For the same MF-composite inhomogeneity and parameters as in Fig. 13 (c) in the "Results" Section of the main paper, we show in Figs. S9 (a), (b), and (c) for, respectively, G_{gap} = 0.5 nS (low coupling), 2 nS (intermediate coupling), and 8 nS (high coupling), pseudocolor plots of V_m, at time t = 2 s, with $\mathcal{G}_{mm}/\mathcal{G}_{mf}$ = 1 and $\mathcal{G}_{mm}/\mathcal{G}_{mf}$ = 200. We also obtain time series for $V_m(x, y, t)$ from a point outside the inhomogeneity (x = 22.5mm, y = 22.5mm) and a point inside it (x = 67.5mm, x = 67.5mm), both of which are depicted by asterisks in Figs. S9 (a)-(c). These time series, with 2×10^5 data points each, are plotted in Figs. S9 (d), (e), and (f) for G_{gap} = 0.5 nS, 2 nS, and 8 nS, respectively (data from the points outside and inside the inhomogeneity are represented, respectively, by black circles and red triangles); Figs. S9 (g), (h), and (i) show the corresponding plots of the ibi versus the beat number n; and the associated power spectra $E(\omega)$ are depicted in Figs. S9 (j), (k), and (l). The Video S10 has four panels that show the spatiotemporal evolution of pseudocolor plots of V_m and the spiral-tip trajectories for 2 s ≤ t ≤ 3 s for a control myocyte layer with no inhomogeneities (top left panel) and, in addition, the simulation domains of Figs. S9 (a)-(c) (top right, bottom left, and bottom right panels). Here too we obtain a rich variety of spiral-wave behaviors inside and outside of the MF-composite inhomogeneity, as in the cases with zero- and single-sided couplings.

References

1. Rook MB, van Ginneken AC, de Jonge B, *et al.* (1992) Differences in gap junction channels between cardiac myocytes, fibroblasts, and heterologous pairs. Am J Physiol 263: C959-C977.

2. Kohl P, Kamkin AG, Kiseleva IS, Noble D (1994) Mechanosensitive fibroblasts in the sino-atrial node region of rat heart: interaction with cardiomyocytes and possible role. Exp Physiol 79: 943-956.

3. Kiseleva I, Kamkin A, Pylaev A, *et al.* (1998) Electrophysiological properties of mechanosensitive atrial fibroblasts from chronic infarcted rat heart. J Mol Cell Cardiol 30: 1083-1093.

4. Kamkin A, Kiseleva I, Wagner KD, *et al.* (1999) Mechanically induced potentials in fibroblasts from human right atrium. Exp Physiol 84: 347-356.

5. Kamkin A, Kiseleva I, Wagner KD, *et al.* (2002) A possible role for atrial fibroblasts in postinfarction bradycardia. Am J Physiol Heart Circ Physiol 282: H842-H849.

6. Chilton L, Ohya S, Freed D, *et al.* (2005) K^+ currents regulate the resting membrane potential, proliferation, and contractile responses in ventricular fibroblasts and myofibroblasts. Am J Physiol Heart Circ Physiol 288: H2931-H2939.

7. Shibukawa Y, Chilton EL, Maccannell KA, *et al.* (2005) K^+ currents activated by depolarization in cardiac fibroblasts. Biophys J 88: 3924-3935.

8. Xie Y, Garfinkel A, Weiss JN, Qu Z (2009) Cardiac alternans induced by fibroblast-myocyte coupling: mechanistic insights from computational models. Am J Physiol Heart Circ Physiol 297: H775-H784.

9. Sachse FB, Moreno AP, Abildskov JA (2008) Electrophysiological modeling of fibroblasts and their interaction with myocytes. Ann BioMed Engg 36: 41-56.

10. Jacquemet V, Henriquez CS (2007) Modelling cardiac fibroblasts: interactions with myocytes and their impact on impulse propagation. Europace 9: vi29-vi37.

11. MacCannell KA, Bazzazi H, Chilton L, *et al.* (2007) A mathematical model of electrotonic interactions between ventricular myocytes and fibroblasts. Biophys J 92: 4121-4132.

12. Jacquemet V (2006) Pacemaker activity resulting from the coupling with nonexcitable cells. Phys Rev E 74: 011908.

13. Miragoli M, Salvarani N, Rohr S (2007) Myofibroblasts induce ectopic activity in cardiac tissue. Circ Res 101: 755-758.

14. Kryukov AK, Petrov VS, Averyanova LS, *et al.* (2008) Synchronization phenomena in mixed media of passive, excitable, and oscillatory cells. Chaos 18: 037129.

15. Majumder R, Nayak AR, Pandit R (2012) Nonequilibrium Arrhythmic States and Transitions in a Mathematical Model for Diffuse Fibrosis in Human Cardiac Tissue. PLoS ONE 7(10): e45040.

16. Shajahan TK, Nayak AR, Pandit R (2009) Spiral-wave turbulence and its control in the presence of inhomogeneities in four mathematical models of cardiac tissue. PLoS ONE 4(3): e4738.

II. Figures

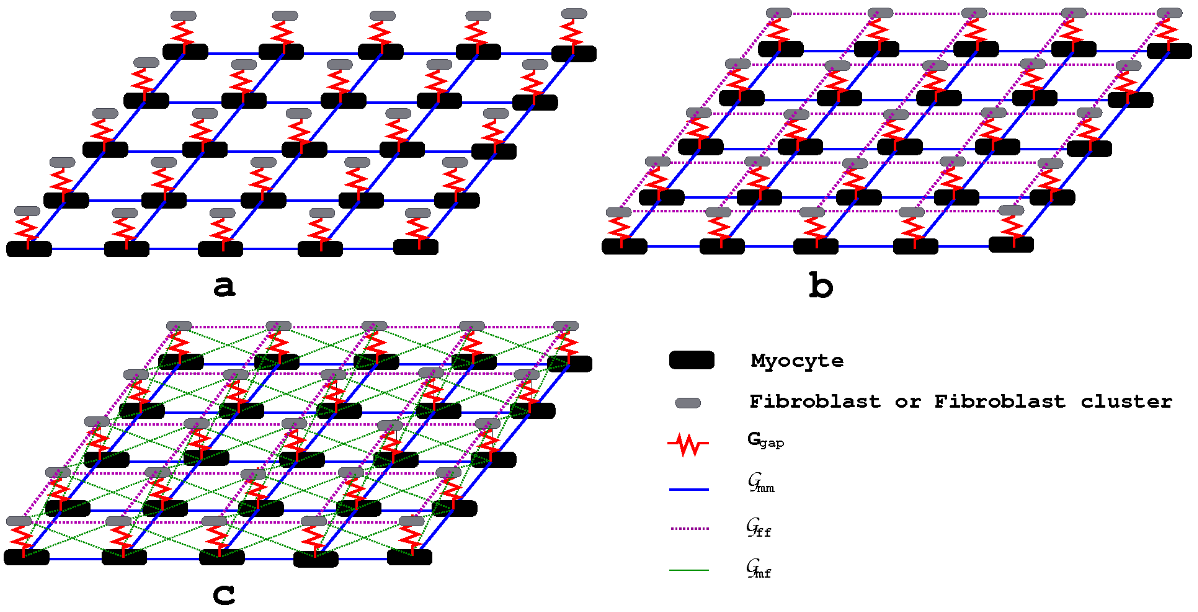

Figure S1. Schematic diagram: A small part of our square simulation domain with sites occupied by myocyte-fibroblast (MF) composites, connected by the gap-junctional conductance G_{gap}, with (a) zero-sided, (b) single-sided, and (c) double-sided couplings between MF composites.

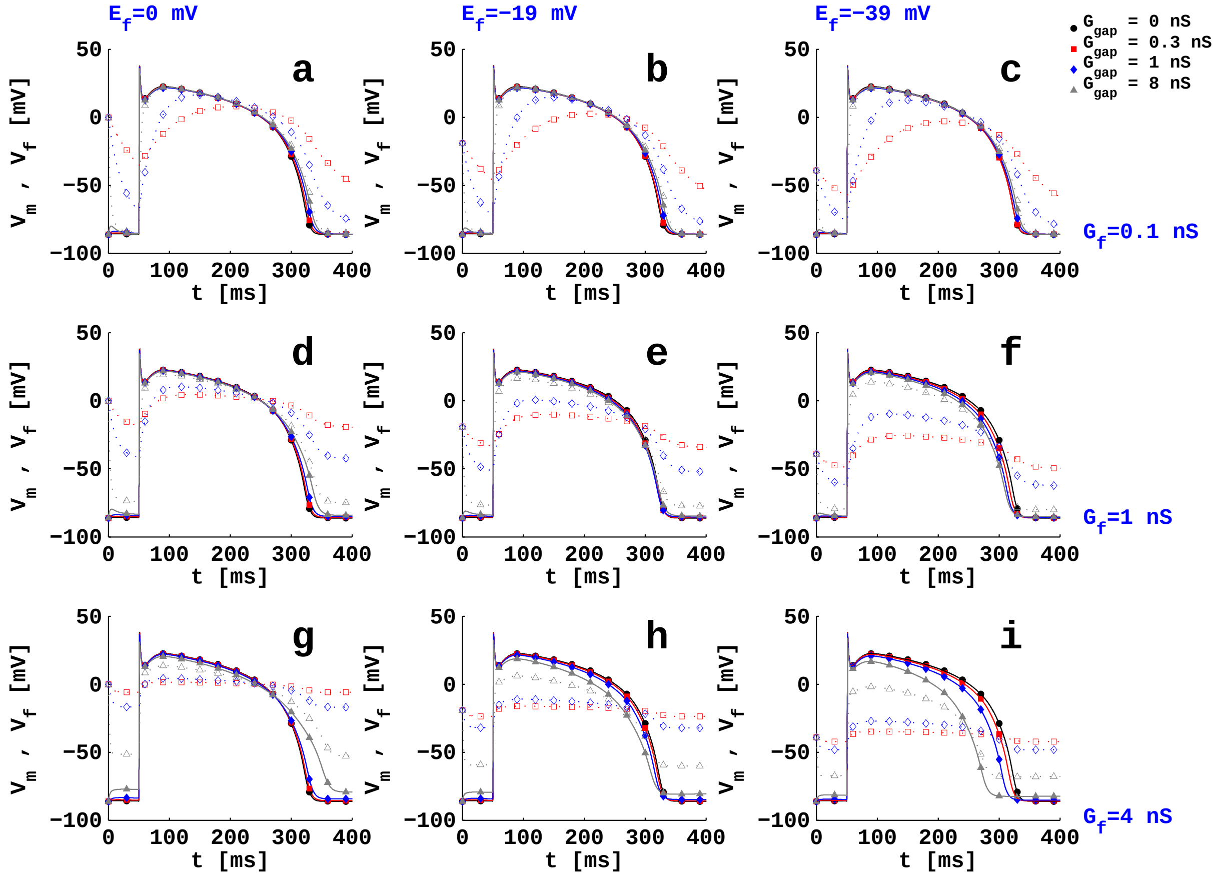

Figure S2. Plots of action potentials: The myocyte action potential V_m (full symbols and lines) and the fibroblast action potential V_f (unshaded symbols and dashed lines), with a passive fibroblast of capacitance $C_{f,tot}$ = 25.2 pF coupled with a myocyte for (a) E_f = 0 mV and G_f = 0.1 nS, (b) E_f = −19 mV and G_f = 0.1 nS, (c) E_f = −39 mV and G_f = 0.1 nS, (d) E_f = 0 mV and G_f = 1 nS, (e) E_f = −19 mV and G_f = 1 nS, (f) E_f = −39 mV and G_f = 1 nS, (g) E_f = 0 mV and G_f = 4 nS, (h) E_f = −19 mV and G_f = 4 nS, and (i) E_f = −39 mV and G_f = 4 nS; red squares (full or unshaded) indicate G_{gap} = 0.3 nS; blue diamonds (full or unshaded) indicate G_{gap} = 1 nS; gray triangles (full or unshaded) indicate G_{gap} = 8 nS; black squares (full or unshaded) indicate an uncoupled myocyte.

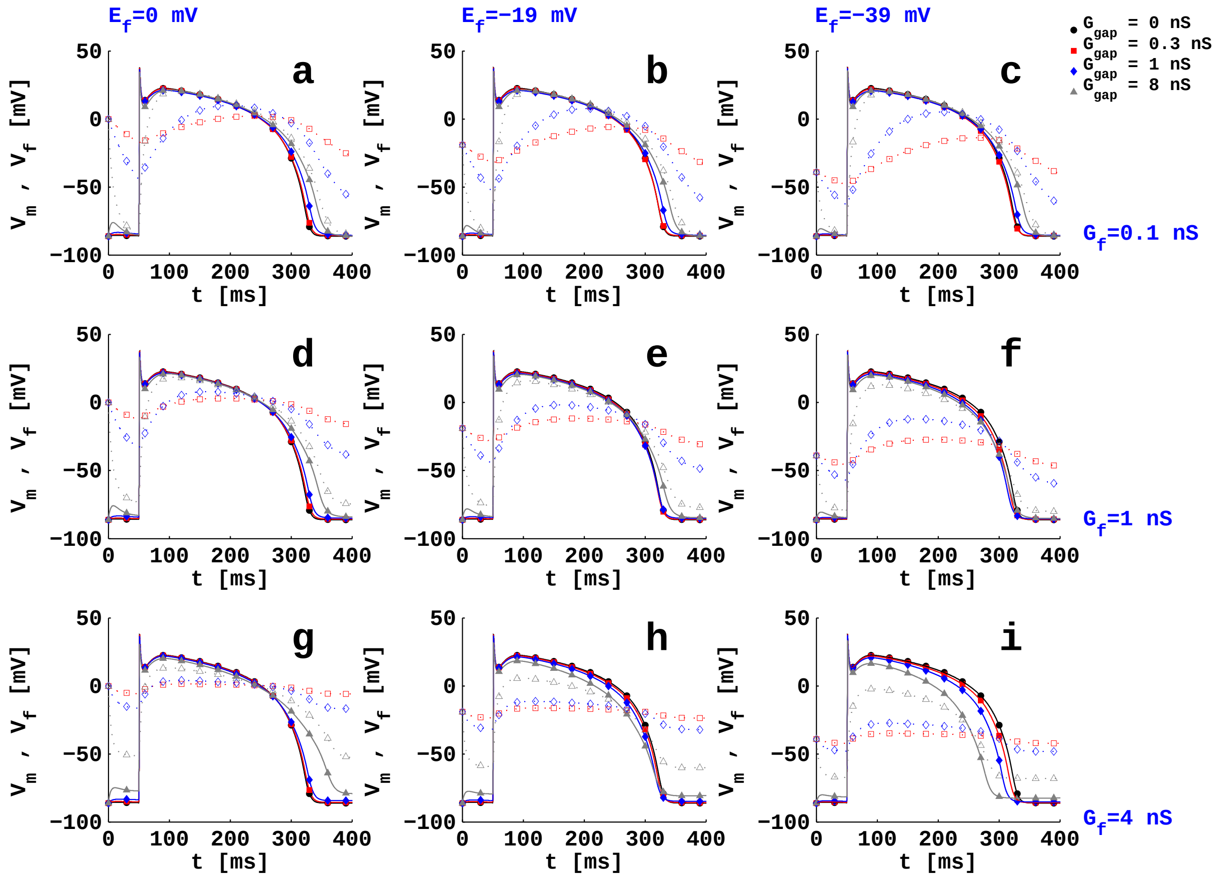

Figure S3. Plots of action potentials: The myocyte action potential V_m (full symbols and lines) and the fibroblast action potential V_f (unshaded symbols and dashed lines), with a passive fibroblast of capacitance $C_{f,tot}$ = 63 pF coupled with a myocyte for (a) E_f = 0 mV and G_f = 0.1 nS, (b) E_f = −19 mV and G_f = 0.1 nS, (c) E_f = −39 mV and G_f = 0.1 nS, (d) E_f = 0 mV and G_f = 1 nS, (e) E_f = −19 mV and G_f = 1 nS, (f) E_f = −39 mV and G_f = 1 nS, (g) E_f = 0 mV and G_f = 4 nS, (h) E_f = −19 mV and G_f = 4 nS, and (i) E_f = −39 mV and G_f = 4 nS; red squares (full or unshaded) indicate G_{gap} = 0.3 nS; blue diamonds (full or unshaded) indicate G_{gap} = 1 nS; gray triangles (full or unshaded) indicate G_{gap} = 8 nS; black squares (full or unshaded) indicate an uncoupled myocyte.

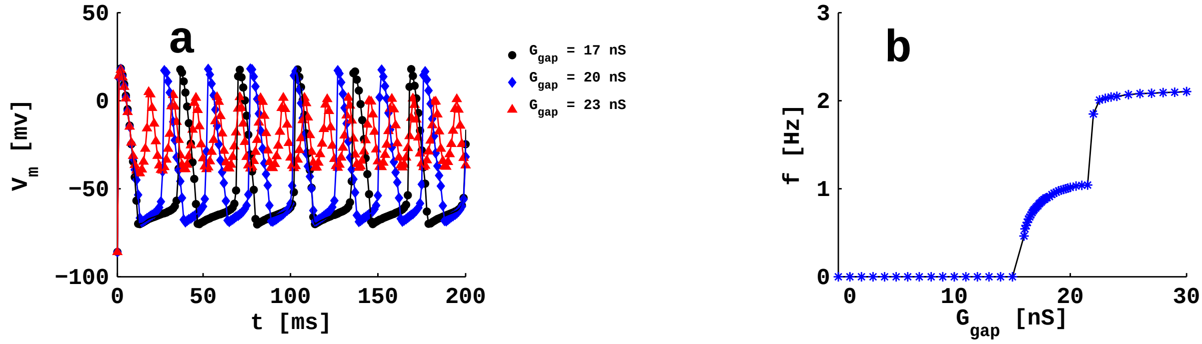

Figure S4. Transmembrane potential of myocyte V_m in an MF composite: (a) Plots of V_m versus t, with $C_{f,tot}$ = 6.3 pF, G_f = 8.0 nS, and E_f = 0 mV for G_{gap} = 17 nS (full black circles), G_{gap} = 20 nS (full blue diamond) and G_{gap} = 23 nS (full red triangle). (b) Plot of the frequency f (see text) versus G_{gap}. The MF composite shows excitable, autorhythmicity and oscillatory behavior in the regime $0 \leq G_{gap} < 16$ nS, $16 \leq G_{gap} < 23$ nS and $G_{gap} \geq 23$ nS, respectively.

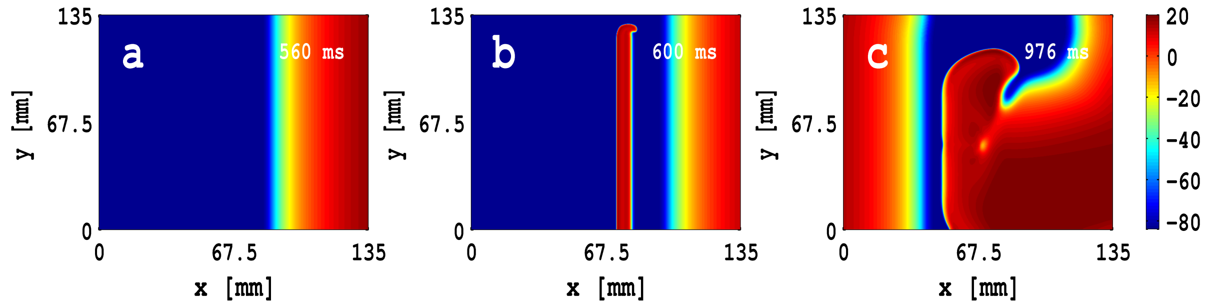

Figure S5. Initiation of a spiral wave by the S1-S2 parallel field protocal: To inject a spiral wave, the diffusion constant is set to D_{mm} = 0.000385 cm^2/ms; this is a quarter of its original value, which is 0.00154 cm^2/ms; an S1 stimulus of strength 150 pA/pF is applied for 3 ms at the left boundary; after 560 ms, an S2 stimulus of strength 450 pA/pF is applied for 3 ms just behind the *refractory tail* of the plane wave initiated by the S1 stimulus; this S2 stimulus is applied over the region $x = 360$ and $1 \leq y \leq 550$. We reset D_{mm} to its original value after 880 ms; this procedure yields a fully developed spiral wave at $t = 976$ ms. The spiral wave configuration in (c) is used as the initial condition for our subsequent studies.

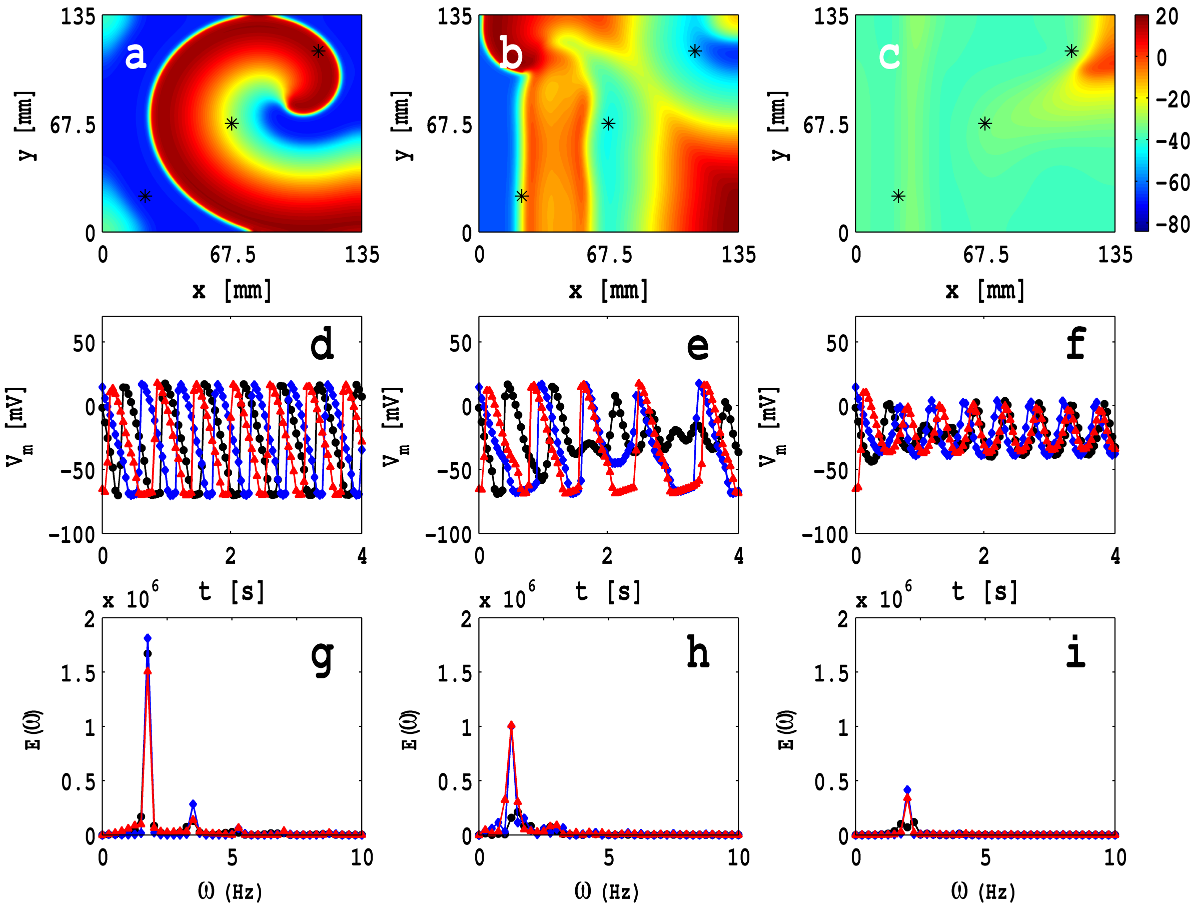

Figure S6. Pseudocolor plots of the myocyte transmembrane potential V_m, at time $t = 2$ s, for the case of zero-sided couplings, with $E_f = 0$ mV and $G_f = 8$ nS: (a) low-frequency autorhythmicity ($G_{gap} = 17$ nS), (b) high-frequency autorhythmicity ($G_{gap} = 20$ nS), and (c) when the MF composite displays oscillatory behavior ($G_{gap} = 23$ nS) (see, Figs. S4 for the boundaries of these regions). Figures (d)-(f) show, respectively, the time series of $V_m(x, y, t)$, in the time interval $0 \text{ s} \leq t \leq 4$ s, obtained from three representative points, shown by asterisks in Fig. S6(a)-(c), namely, ($x = 22.5$ mm, $y = 22.5$ mm) (black filled circles), ($x = 67.5$ mm, $y = 67.5$ mm) (blue filled diamonds), and ($x = 112.5$ mm, 112.5 mm) (red filled triangles). Figures S6(g)-(i) show the corresponding power spectra, which we calculate from these time series, each of which has 2×10^5 data points.

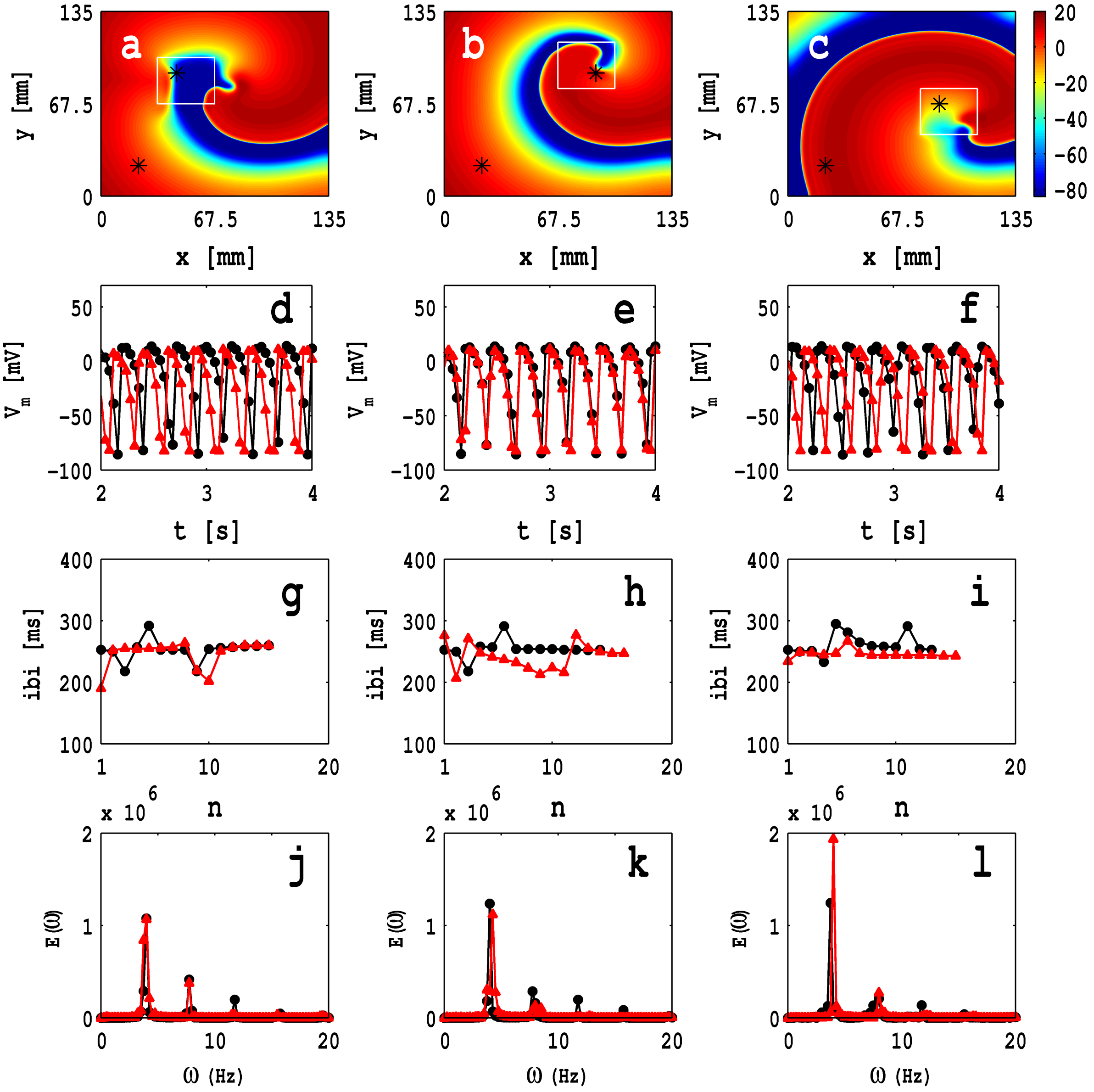

Figure S7. Pseudocolor plots of the transmembrane potential V_m of the myocyte at time, $t = 2$ s, in the presence of a square MF-composite inhomogeneity, of side $\ell = 33.75$ mm, for the case of single-sided coupling with $\mathcal{G}_{mm}/\mathcal{G}_{mf} = 1$; the bottom-left corner of the inhomogeneity is fixed at (a) ($x = 33.75$ mm, $y = 67.5$ mm), (b) ($x = 56.25$ mm, $y = 56.25$ mm), and (c) ($x = 78.75$mm, $y = 45$mm); the white solid lines in these figures show the spiral-tip trajectories in the time interval 2 s $\leq t \leq 3$ s and the local time series data are recorded from points that are shown by asterisks. The plots in (d)-(f) show the time series for V_m, in the interval 0 s $\leq t \leq 4$ s, which are obtained from the points outside (($x = 112.5$mm, $y = 112.5$mm) for all cases) and inside the fibroblast inhomogeneity (($x = 45$mm, $y = 90$mm), ($x = 67.5$mm, $y = 67.5$mm), and ($x = 90$mm, $y = 67.5$mm) for (a), (b), and (c), respectively), represented by black circles and red triangles, respectively; (g), (h), and (i) show plots of the inter-beat intervals (ibis) versus the beat number n for the time series of V_m mentioned above; each one of these time series contain 2×10^5 data points; the power spectra $E(\omega)$, which follow from these time series, are given in (j), (k), and (l).

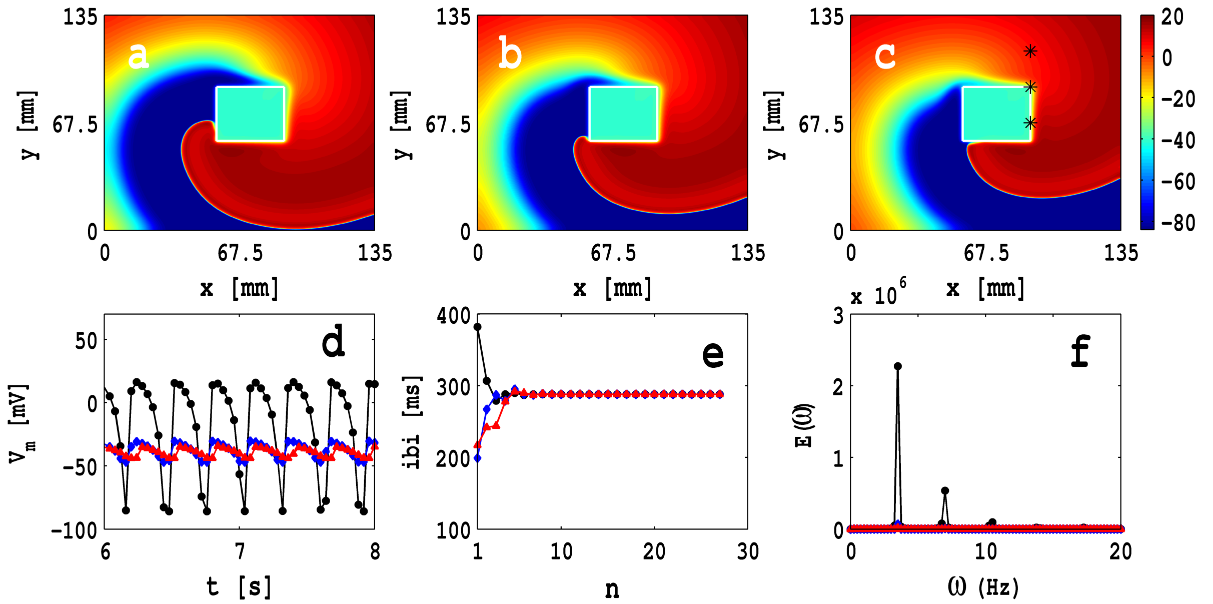

Figure S8. Pseudocolor plots of V_m with an MF-compositie inhomogeneity: The transmembrane potential of the myocyte V_m at time, (a) $t = 2$ s, (b) $t = 4$ s, and (c) $t = 6$ s, when a square MF-composite inhomogeneity with, $\mathcal{G}_{mm}/\mathcal{G}_{ff} = 1$ and $\mathcal{G}_{mm}/\mathcal{G}_{mf} = 1$, and of length $\ell = 33.75$mm, is placed with its lower-left corner at $(x = 67.5\text{mm}, y = 67.5\text{mm})$; (d) shows the time series of $V_m(x, y, t)$, in the time interval $0 \text{ s} \leq t \leq 4$ s, obtained from three representative points, shown by asterisks in (c), namely, $(x = 90 \text{ mm}, y = 112.5 \text{ mm})$ (black filled circles), $(x = 90 \text{ mm}, y = 90 \text{ mm})$ (blue filled diamonds), and $(x = 90 \text{ mm}, 67.5 \text{ mm})$ (red filled triangles); the corresponding ibi's, calculated from data sets of 4×10^5 points, are shown in (e); (f) shows the power spectrum of the above time series of V_m of length 2×10^5 data points (after removing the initial 2×10^5 iteration steps).

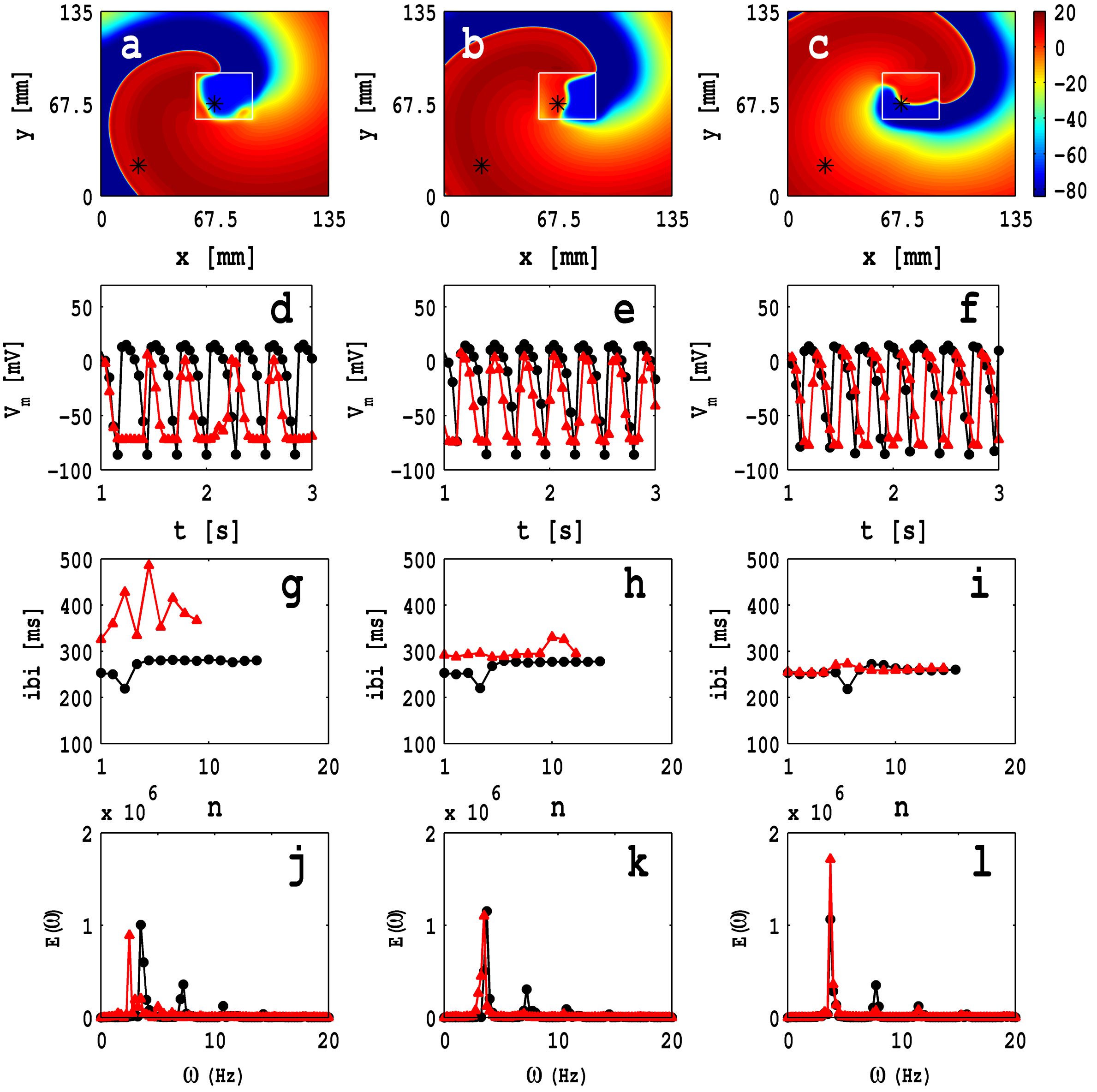

Figure S9. Pseudocolor plots of the transmembrane potential of the myocyte V_m, at time $t = 2$ s, in the presence of a square, MF-composite inhomogeneity, of side $\ell = 33.75$ mm and with its lower-left-hand corner placed at $(x = 56.25$ mm$, y = 56.25$ mm$)$ for the case of doubled-sided coupling with $\mathcal{G}_{mm}/\mathcal{G}_{ff} = 1$ and $\mathcal{G}_{mm}/\mathcal{G}_{ff} = 200$: (a) $G_{gap} = 0.5$ nS, (b) $G_{gap} = 2$ nS, and (c) $G_{gap} = 8$ nS. Video S10 illustrates the spatiotemporal evolution of these spiral waves. The time series for $V_m(x, y, t)$, with 2×10^5 data points each, from a point outside the inhomogeneity $(x = 22.5$mm$, y = 22.5$mm$)$ and a point inside it $(x = 67.5$mm$, x = 67.5$mm$)$, both of which are depicted by asterisks in (a)-(c), are plotted in (d), (e), and (f) for $G_{gap} = 0.5$ nS, 2 nS, and 8 nS, respectively (data from the points outside and inside the inhomogeneity are represented, respectively, by black circles and red triangles); (g), (h), and (i) show the corresponding plots of the ibi versus the beat number n; and the associated power spectra $E(\omega)$ are depicted in (j), (k), and (l).

$C_{f,tot}$ (pA)	G_f (nS)	G_{gap} (nS)	E_f (mV)	N_f	$\Delta APD_{70\%}$ (ms)	$\Delta APD_{80\%}$ (ms)	$\Delta APD_{90\%}$ (ms)	$\Delta \dot{V}_{max}$ (mV/ms)	ΔV_{rest} (mV)
6.3	0.1	0.3	-9	1	0.82	0.90	1.06	-2.41	0.12
6.3	0.1	0.3	-9	2	1.66	1.82	2.14	-5.49	0.25
6.3	0.1	0.3	-9	3	2.52	2.78	3.26	-9.28	0.38
6.3	0.1	0.3	-9	4	3.42	3.76	4.42	-13.83	0.52
6.3	0.1	0.3	-9	5	4.32	4.76	5.62	-19.16	0.67
6.3	0.1	0.3	-9	6	5.26	5.80	6.86	-22.21	0.82
6.3	0.1	0.3	-9	7	6.22	6.86	8.12	-25.40	0.99
6.3	0.1	0.3	-9	8	7.22	7.96	9.44	-29.41	1.16
6.3	0.1	0.3	-9	9	8.24	9.08	10.80	-34.21	1.34
6.3	0.1	0.3	-9	10	9.28	10.24	12.20	-39.74	1.52
6.3	0.1	0.3	-9	11	10.36	11.44	13.64	-45.68	1.72
6.3	0.1	0.3	-9	12	11.48	12.66	15.14	-48.79	1.92
6.3	0.1	0.3	-19	1	0.26	0.34	0.50	-2.08	0.10
6.3	0.1	0.3	-19	2	0.56	0.70	1.00	-4.64	0.21
6.3	0.1	0.3	-19	3	0.86	1.08	1.54	-7.72	0.33
6.3	0.1	0.3	-19	4	1.18	1.50	2.12	-11.34	0.45
6.3	0.1	0.3	-19	5	1.52	1.92	2.70	-15.53	0.57
6.3	0.1	0.3	-19	6	1.86	2.36	3.34	-20.29	0.70
6.3	0.1	0.3	-19	7	2.24	2.84	3.98	-22.61	0.84
6.3	0.1	0.3	-19	8	2.64	3.32	4.68	-25.34	0.98
6.3	0.1	0.3	-19	9	3.06	3.84	5.40	-28.67	1.13
6.3	0.1	0.3	-19	10	3.50	4.38	6.16	-32.58	1.28
6.3	0.1	0.3	-19	11	3.96	4.94	6.94	-37.04	1.44
6.3	0.1	0.3	-19	12	4.44	5.52	7.78	-41.99	1.60
6.3	0.1	0.3	-29	1	-0.28	-0.22	-0.08	-1.76	0.09
6.3	0.1	0.3	-29	2	-0.54	-0.40	-0.12	-3.85	0.18
6.3	0.1	0.3	-29	3	-0.80	-0.58	-0.16	-6.29	0.28
6.3	0.1	0.3	-29	4	-1.04	-0.76	-0.18	-9.10	0.37
6.3	0.1	0.3	-29	5	-1.28	-0.90	-0.18	-12.29	0.48
6.3	0.1	0.3	-29	6	-1.50	-1.04	-0.14	-15.89	0.58
6.3	0.1	0.3	-29	7	-1.70	-1.16	-0.08	-19.87	0.69
6.3	0.1	0.3	-29	8	-1.88	-1.26	0.02	-22.37	0.81
6.3	0.1	0.3	-29	9	-2.04	-1.32	0.10	-24.49	0.93
6.3	0.1	0.3	-29	10	-2.20	-1.38	0.24	-27.02	1.05
6.3	0.1	0.3	-29	11	-2.32	-1.42	0.40	-29.96	1.18
6.3	0.1	0.3	-29	12	-2.44	-1.44	0.60	-33.30	1.31
6.3	0.1	0.3	-39	1	-0.82	-0.76	-0.64	-1.45	0.07
6.3	0.1	0.3	-39	2	-1.64	-1.52	-1.26	-3.11	0.15
6.3	0.1	0.3	-39	3	-2.46	-2.26	-1.86	-4.99	0.22
6.3	0.1	0.3	-39	4	-3.26	-2.98	-2.46	-7.10	0.30
6.3	0.1	0.3	-39	5	-4.04	-3.70	-3.02	-9.45	0.39
6.3	0.1	0.3	-39	6	-4.82	-4.40	-3.58	-12.05	0.47
6.3	0.1	0.3	-39	7	-5.58	-5.08	-4.10	-14.90	0.56
6.3	0.1	0.3	-39	8	-6.32	-5.74	-4.62	-18.00	0.65
6.3	0.1	0.3	-39	9	-7.04	-6.40	-5.10	-21.36	0.74
6.3	0.1	0.3	-39	10	-7.76	-7.04	-5.56	-23.17	0.83
6.3	0.1	0.3	-39	11	-8.46	-7.64	-5.98	-24.89	0.93
6.3	0.1	0.3	-39	12	-9.14	-8.24	-6.38	-26.87	1.03

Table S1. The values of $C_{f,tot}$, G_f, G_{gap}, E_f, and N_f for a single MF composite and the changes in the AP morphology, relative to that of an uncoupled myocyte. We concentrate on the APD, $\dot{V}_{max}$, and V_{rest} and list the changes, indicated by Δ, in these parameters. $\Delta APD_{70\%}$, $\Delta APD_{80\%}$, and $\Delta APD_{90\%}$ denote, respectively, the changes in the APD at 70%, 80%, and 90% repolarization. Note that here we have low values (see text) for both G_f (0.1 nS) and G_{gap} (0.3 nS).
